# Genome-scale modeling of Chinese hamster ovary cells by hybrid semi-parametric flux balance analysis

**DOI:** 10.1007/s00449-022-02795-9

**Published:** 2022-10-16

**Authors:** João R. C. Ramos, Gil P. Oliveira, Patrick Dumas, Rui Oliveira

**Affiliations:** 1 LAQV REQUIMTE, Department of Chemistry, NOVA School of Science and Technology, NOVA University Lisbon, Campus Caparica, 2829-516 Caparica, Portugal; 2 GSK, 89 rue de l’Institut, 1330 Rixensart, Belgium

**Keywords:** Genome-scale modeling, CHO-K1 cells, Flux balance analysis, Hybrid semi-parametric systems, Machine learning, Culture media design

## Abstract

Flux balance analysis (FBA) is currently the standard method to compute metabolic fluxes in genome-scale networks. Several FBA extensions employing diverse objective functions and/or constraints have been published. Here we propose a hybrid semi-parametric FBA extension that combines mechanistic-level constraints (parametric) with empirical constraints (non-parametric) in the same linear program. A CHO dataset with 27 measured exchange fluxes obtained from 21 reactor experiments served to evaluate the method. The mechanistic constraints were deduced from a reduced CHO-K1 genome-scale network with 686 metabolites, 788 reactions and 210 degrees of freedom. The non-parametric constraints were obtained by principal component analysis of the flux dataset. The two types of constraints were integrated in the same linear program showing comparable computational cost to standard FBA. The hybrid FBA is shown to significantly improve the specific growth rate prediction under different constraints scenarios. A metabolically efficient cell growth feed targeting minimal byproducts accumulation was designed by hybrid FBA. It is concluded that integrating parametric and nonparametric constraints in the same linear program may be an efficient approach to reduce the solution space and to improve the predictive power of FBA methods when critical mechanistic information is missing.

**Supplementary Information:**

The online version contains supplementary material available at 10.1007/s00449-022-02795-9.

## Introduction

Genome-scale models (GEMs) are systems-level representations of the entirety of metabolic functions of a cell [1]. They are reconstructed from the full set of annotated gene-to-protein relationships (GPRs). GEMs undergo several curation steps whereby reactions, metabolites and GPRs are reviewed using preferably standardized procedures [2]. After careful curation, GEM reactions eventually become mass-, charge- and energy-balanced with sufficient quality for stoichiometric balancing. GEMs give rise to typically large, sparse and rank deficient stoichiometric matrices. Assuming balanced intracellular metabolite pools, undetermined systems of linear algebraic equations are obtained. Flux Balance Analysis (FBA) became the standard method to compute metabolic fluxes in GEMs under the hypothesis of a metabolic objective [3]. Standard FBA applies linear programming (LP) to compute metabolic fluxes under a pre-defined objective function, assuming reaction stoichiometry constraints, balanced intracellular metabolite pools (steady-state hypothesis) and flux irreversibility constraints [4, 5]. Dynamic FBA (dFBA) extensions have been applied to GEMs to compute metabolic fluxes over time using minimal kinetic information [6]. Due to the undetermined nature of the constraints, FBA solutions are typically not unique, with many alternative optima achieving the same objective. Flux Variability Analysis (FVA) is widely used for evaluating alternative optima. FVA computes the minimum and maximum range of each reaction flux that can still satisfy the constraints [7]. Many other FBA extensions employing different constraints, objective functions and LP implementations have been proposed (see review by Anand et al. [8]).

The first published consensus GEM of CHO cells is relatively recent [9]. It comprehends 6663 reactions and 4456 metabolites, particularized in 3 cell line variants (CHO-K1, CHO-DG44 and CHO-S). Since the publication of this resource, a few studies have attempted to use GEMs to optimize CHO cell culture employing different FBA techniques. Hong et al*.* applied standard FBA to study the effect of sparging conditions on CHO-DG44 [10]. The FBA was constrained by measured fluxes of amino acids, glucose, lactate, specific growth rate and specific antibody productivity. It was found that mild and harsh sparging conditions lead to decreased cell growth, viability and productivity [10]. The authors concluded that sparging stress rewires amino acid metabolism towards H_2_O_2_ turnover, thus they hypothesized that increased amino acid uptake caused by sparging stress contributes to restoring the redox homeostasis against oxidative stress.

Yeo et al*.* expanded the previously published CHO GEM (changes in pathways such as cholesterol metabolism, fatty acid activation, elongation and desaturation, glycerophospholipid metabolism, and *N*- and *O*-glycan biosynthesis) and added enzyme capacity constraints within the flux balance analysis framework (ecFBA) to significantly reduce the flux variability in a biologically meaningful manner [11]. This allowed for good prediction of lactate metabolism for different CHO clones grown on different media. They concluded that the lactate-pyruvate cycling could be beneficial for CHO cells to efficiently utilize the mitochondrial redox capacity and that ecFBA could be used to identify key engineering targets.

Calmels and co-authors manually curated and reduced the CHO-DG44 GEM by modifying 601 reactions [12]. These modifications were intended to simplify the model and to cope with missing constraints related to regulatory effects as well as thermodynamic and osmotic forces. The parsimonious enzyme usage FBA (pFBA) method was employed constrained by the uptake and secretion of 24 metabolites. The objective function was the maximization of cell growth. They showed that the reduced GEM allowed good predictions of extracellular metabolites rates (*r*^2^ ≥ 0.8) and good prediction of cell growth rate (*r*^2^ = 0.91). This study highlights the adaption of a CHO GEM to an industrial process.

Recently, Schinn and co-authors combined a modified CHO GEM with a statistical learning method for time-course prediction of individual amino acid concentrations in fed-batch cultivations of 10 CHO clones with different growth and productivity profiles [13]. The statistical learning feature of the model consisted in two empirically derived equations that ‘offsets’ flux predictions by FBA. Overall, this approach allowed for good approximation of most amino acid consumptions [excluding alanine (Ala) and glycine (Gly)], when the steady-state assumption holds true. They suggested the use of this approach to control nutrients feeding to avoid premature nutrient depletion or to provide early predictions of failed bioreactor runs.

Metabolic models with different levels of detail have been extensively used for CHO culture media design [14]. Fouladiha et al. [15] used the iCHO1766 GEM [9] to identify key medium components to increase monoclonal antibody production by CHO cells. Huang and co-authors performed culture medium optimization using the CHO-K1 full GEM targeting IgG production improvement [16]. Standard FBA was applied to calculate optimal flux scenarios in the pre-induction phase (maximization of the specific growth rate) and in the post-induction phase (maximization IgG specific productivity) for two different media and the same cell line (CHO-K1 GS knockout). They analyzed the metabolic differences between these two cell culture conditions by metabolic pathway analysis. Through the comparison of pathway fold-change between high and low production cases, they have hypothesized culture medium enrichment scenarios. They successfully increased IgG productivity by 33% by enriching the feed with 3 amino acids [i.e., Leucine (Leu), Isoleucine (Ile) and Valine (Val)]. It should be noted that the CHO-K1 GEM was not used as a predictive tool in this study. It was rather used as a tool to generate rational hypothesis for culture media improvement.

As shown in the literature review, different FBA extensions with diverse constraints were applied to CHO GEMs. Standard FBA uses limited fundamental assumptions, namely the reactions stoichiometry, the pseudo steady-state hypothesis and the thermodynamic reaction directionality. Adding “realistic” constraints may in principle reduce the solution space and improve FBA predictions. A few recent studies have proposed hybrid methods that combine FBA with empirical modeling techniques [17]. Vijayakumar and co-authors proposed a hybrid methodology that integrates FBA, GEMs, multi-omic data and machine learning to refine phenotypic predictions [18]. Sahu et al. have recently reviewed approaches to extend FBA with machine learning techniques [19]. Hong et al. developed a framework for media design based on a CHO-GEM, FBA and multivariate data analysis [20]. In previous studies, we have introduced hybrid metabolic flux analysis [21, 22] and hybrid elementary modes analysis [23, 24]. Following this line of development, we propose here a hybrid semi-parametric FBA extension (HybridFBA). HybridFBA formally combines parametric constraints (derived from mechanistic knowledge) with nonparametric constraints (derived from data) in the same LP. More specifically, HybridFBA extends standard FBA by adding flux correlation constraints deduced by Principal Component Analysis (PCA) of experimental flux data. HybridFBA computes simultaneously “mechanistic” decision variables (fluxes) and empirical decision variables (scores of principle components) in a single LP. Measured flux data typically contains valuable information of unknown regulatory mechanisms. As shown in the results section, the inclusion of such empirical constraints in HybridFBA significantly improves metabolic flux predictions.

## Materials and methods

### Cell culture and analytics

Pre-cultures of a GSK proprietary CHO-K1 cell line coding for a target antigen were grown in shake-flasks (Corning, NY, USA) at 37 °C. Cells were cultivated in a GSK proprietary chemically defined, protein-free and animal component-free medium.

In total, 21 cell cultures for antigen production were carried out in in 5 or 10 L glass vessels (Sartorius, Göttingen, Germany), with an initial seed of 0.4–1.0 Mcell/mL. The pH was controlled at 7.0 with 0.5 M NaOH and sparged CO_2_ together with overlay aeration. DO was controlled at 30% by sparging pure oxygen. Stirring was adjusted to around 20 W/m^3^ in all scales of the culture vessel. Once a high enough viable cell density was reached, the temperature was decreased to 33 °C, causing growth to gradually stop and the antigen production to start (temperature-shift induction). Depending on the seeding density and on the target biomass at temperature shift, the whole cell culture lasted for 12–17 days.

The base medium was the same in all cultivations, but the feeding solutions changed from one culture to another during the fed-batch operation mode. The feeding solutions consisted in mixtures of amino acids, glucose and pyruvate. Feeding happened once a day and consisted in the quasi-simultaneous addition of a bolus of all the feeding solutions involved.

Sampling was performed daily (with some exceptions). Viable cell density and viability were assayed using a Vi-Cell cell counter (Beckman, Indianapolis, USA). Glucose (Glc), lactate (Lac), pyruvate (Pyr), glutamine (Gln), ammonium (NH4), glycerol (Glyc) and lactate dehydrogenase (LDH) were assayed using a CedexBio-HT metabolite analyzer (Roche, Penzberg, Germany). The antigen quantification was performed off-line with an Octet HTX (Pall, NY, USA). The remaining metabolites and amino acids were assayed off-line by Nuclear Magnetic Resonance spectroscopy (NMR) at Eurofins Spinnovation (Oss, The Netherlands).

### Extracellular reaction rates estimation and analysis

Steady-state reaction rates of 27 extracellular species were estimated for the exponential growth phase (approximately 0–70 cultivation hours) of 21 independent reactor experiments. The reaction rates*,* with units mmol/(g-DW×h), were obtained by robust linear regression of formed quantity (units of mmol) against the integral of viable cell mass (units of g-DW×h). Gln is chemically unstable decomposing with first-order kinetics into equimolar quantities of pyrrolidone carboxylic acid and NH4 [25, 26]. The total amounts of Gln and NH4 that resulted from extracellular decomposition were subtracted to the total formed quantity before the respective rates were estimated. Details of the rates estimation method are provided in the supplementary material.

The resulting flux data, comprising 21 data points (rows) and 27 measured fluxes (columns), were auto-scaled column wise to zero mean and unit variance. The normalized data were subject to principal component analysis (PCA). PCA is a dimensionality-reduction technique that is used to transform a large set of highly correlated variables (in the present case the 27 measured fluxes) into a smaller set of uncorrelated variables [Principal Components (PCs)] while retaining most of the information of the original set. Mathematically, PCA performs the following space transformation

1 $${\overline{r}}_{\mathrm{exch}}={\mathrm{Scores}\times \mathrm{Coeff}}^{T}$$

with ${\overline{r}}_{\mathrm{exch}}$ the normalized rate data, $\mathrm{Coeff}$ the matrix of loadings and $\mathrm{Scores}$ the values of PCs in the transformed data space. The PCs are new transformed variables constructed as linear combinations of the original variables. The resulting PCs are orthogonal thus uncorrelated. The *i*th column of the $\mathrm{Coeff}$ matrix contains the coefficients that linearly transform the original variables into the *i*th PC. For an effective dimensionality reduction, the number of PCs $(\mathrm{NPC})$ should be much smaller than the number or original variables (27 fluxes in the present problem). On the other hand, NPC should be large enough to capture most of the information of the original data. In the present study, NPC was chosen to capture at least 90% of explained variance of the original flux data. The standard singular value decomposition algorithm was employed (MATLAB implementation, function *pca*, algorithm ‘svd’).

### CHO genome-scale model (GEM)

The consensus CHO-K1 GEM [9], accessible in www.chogenome.org [27], was adopted in this study. This model contains 2773 metabolites, 4723 reactions and 2603 degrees of freedom (the number of degrees of freedom corresponds to the difference between the number of reactions and the rank of the stoichiometric matrix of intracellular species). A reduction was performed based on previously published methodologies [28–30]. Particularly, the CHO-K1 model transport reactions were reduced to match GSK proprietary medium composition. This implied the automatic elimination of 3935 intracellular reactions to maintain consistency. This process resulted in a reduced GEM, which is medium specific, containing 627 intracellular metabolites, 788 reactions and all the required extracellular species to match GSK proprietary medium composition. Details of the reduction process are provided in the supplementary material. The reduced model is translated into the following system of linear algebraic equations:

2a $$0={S}_{\mathrm{int}}v$$

2b $${\mathrm{LB}}^{v}\le v\le {\mathrm{UB}}^{v}$$

2c $${r}_{\mathrm{exch}}={S}_{\mathrm{ext}}v$$

with ${S}_{\mathrm{int}}$ the (627 × 788) intracellular stoichiometric matrix, $v$ the (788 × 1) flux vector, ${\mathrm{LB}}^{v}$ and ${\mathrm{UB}}^{v}$ are the lower and upper bound limits of $v$ (the reduced model has 478 irreversible reaction with ${\mathrm{LB}}^{v}=0), {S}_{\mathrm{ext}}$ is the (next × 788) extracellular stoichiometric matrix (the variable next refers to the number of extracellular metabolites in the reduced model), ${r}_{exch}$ the net exchange flux vector with dimension (nrexch × 1) (nrexch refers to the total number of exchange reactions in the reduced model). This system is highly undetermined with 210 degrees of freedom [difference between 788 and the $rank({S}_{\mathrm{int}})$]. The validity of the model was confirmed by linear least squares regression of computed against measured extracellular fluxes for each experiment individually. The sum of squared residuals was negligible in all cases.

### Hybrid semi-parametric flux balance analysis (HybridFBA)

**Fig. 1:**
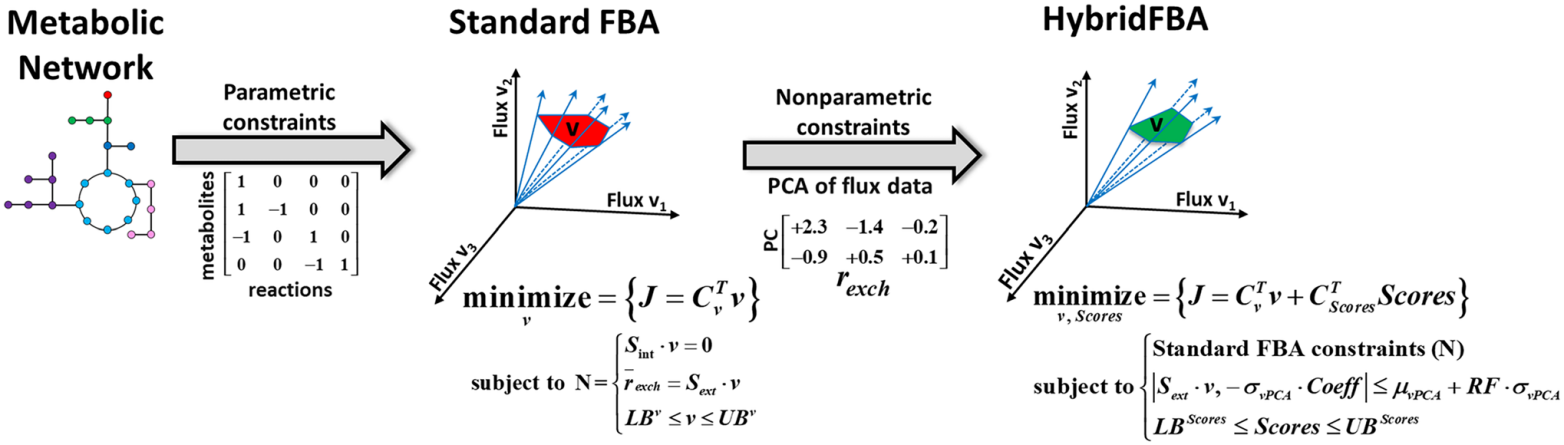
Schematic representation of Hybrid semi-parametric Flux Balance Analysis (HybridFBA)

HybridFBA is an extension of FBA that incorporates mechanistic constraints (parametric) and empirical constraints (nonparametric) in the same LP (Fig. 1). The general principle is to reduce the solution space by combining parametric and nonparametric constraints. The parametric constraints are set by the metabolic network [Eqs. (2a, b, c)]. This part is the same as standard FBA. The nonparametric constraints are obtained from the PCA of experimental flux data [Eq. (1)]. Each column of the $\mathrm{Coeff}$ matrix defines a PC. Each PC imposes a hard constraint on the direction of variation of groups of fluxes. PC constraints need to be compatible with the stoichiometric constraints. Combining Eq. (2c) with Eq. (1) results in the following equation:

3 $${\mu }_{\mathrm{vPCA}}=\left[\begin{array}{cc}{S}_{\mathrm{ext}},& -{\sigma }_{\mathrm{vPCA}}\circ \mathrm{Coeff}\end{array}\right]\left[\begin{array}{c}v\\ \mathrm{Scores}\end{array}\right]$$

with ${\mu }_{\mathrm{vPCA}}$ and ${\sigma }_{\mathrm{vPCA}}$ the mean and standard deviation vectors used for the auto-scaling in the PCA and $\circ$ the Hadamard multiplication. Equation (3) states that the ${r}_{\mathrm{exch}}$ vector calculated mechanistically and empirically must match each other. The PCA model [Eq. (1)] is typically corrupted by error with the explained variance of flux data < 100%. Depending on the PCA error, obeying to Eq. (3) might be infeasible. As such, Eq. (3) was relaxed to inequality constraints under the control of the relaxation factor (RF):

4 $${\mu }_{\mathrm{vPCA}}-\mathrm{RF}{\sigma }_{\mathrm{vPCA}}\le \left[\begin{array}{cc}{S}_{\mathrm{ext}},& -{\sigma }_{\mathrm{vPCA}}\circ \mathrm{Coeff}\end{array}\right]\left[\begin{array}{c}v\\ \mathrm{Scores}\end{array}\right]\le {\mu }_{\mathrm{vPCA}}+\mathrm{RF}{\sigma }_{\mathrm{vPCA}}$$

In practice, Eq. (4) constraints the direction of variation of exchange fluxes imposed by the columns of the $\mathrm{Coeff}$ matrix (e.g., the *i*th column of the $\mathrm{Coeff}$ matrix defines de fluxes direction of *i*th PC.

Upper and lower bounds on the exchange rates [Eq. (5)] were also defined to constraint the LP to a desired interval ${r}_{\mathrm{mean}}\pm k\sigma$

5 $${r}_{\mathrm{mean}}-k\sigma \le \left[\begin{array}{cc}{S}_{\mathrm{ext}},& 0\end{array}\right]\left[\begin{array}{c}v\\ \mathrm{Scores}\end{array}\right]\le {r}_{\mathrm{mean}}+k\sigma$$

with ${r}_{\mathrm{mean}}$ the mean measured rate among the 21 experiments (Table 1), $\sigma$ the standard deviation of measured rates among the 21 experiments (Table 1) and $k>0$ a parameter set by the user to shrink/enlarge the design space. In the results section, k was varied between 1 and 6 to assess the predictive power of HybridFBA in different test case scenarios.

**Table 1:** Mean flux values along with their respective standard deviation and coefficient of variation during exponential growth aggregated from 21 independent cultivations

	This study			Literature data	
Name	Mean	S.D	CV	(1)	(2)
µ	2.82E–02	3.36E-03	11.9	3.06E–02	2.47E–02
Glc	− 3.84E–01	1.05E–01	27.3	− 2.71E–01	− 1.98E–01
Lac	2.56E–01	1.41E–01	55.1	1.36E–01	1.21E–01
Gln	− 7.37E–02	3.31E–02	44.9	2.77E–03	− 6.74E–02
Glu	8.90E–03	4.84E–03	54.4	− 1.24E–02	9.50E–03
NH4	5.92E–02	2.82E–02	47.6	4.38E–02	8.33E–02
Pyr	2.04E–03	2.00E–02	980	− 1.08E–02	NA
Glyc	5.63E–03	1.57E–03	27.9	1.51E–02	NA
Cit	– 9.41E–04	1.96E–03	208	2.21E–03	NA
Ala	9.47E–02	1.21E–02	12.8	2.37E–02	1.13E–02
Arg	− 1.09E–02	4.81E–03	44.1	− 1.10E–02	− 2.01E–02
Asn	− 7.13E–02	1.19E–02	16.7	− 7.47E–02	− 4.06E–02
Asp	3.11E–03	5.30E–03	170	− 2.52E–02	− 9.76E–03
LCystin	− 2.74E–03	2.64E–03	96.4	− 5.22E–03	− 5.39E–03
Gly	2.49E–02	4.37E–03	17.6	6.97E–03	2.09E–02
His	− 5.62E–03	1.90E–03	33.8	− 6.46E–03	− 4.07E–03
Ile	− 1.37E–02	5.42E–03	39.6	− 1.18E–02	− 1.06E–02
Leu	− 2.13E–02	6.59E–03	30.9	− 2.02E–02	− 1.57E–02
Lys	− 9.82E–03	6.15E–03	62.6	− 1.26E–02	− 1.40E–02
Met	− 4.77E–03	4.28E–03	89.7	− 4.80E–03	− 6.32E–03
Phe	− 6.41E–03	3.13E–03	48.8	− 6.46E–03	− 5.92E–03
Pro	− 5.00E–03	6.36E–03	127	– 1.16E–02	–8.47E–03
Ser	− 3.69E–02	9.66E–03	26.2	− 4.31E–02	− 4.81E–02
Thr	− 1.03E–02	5.76E–03	55.9	− 1.53E–02	− 1.06E–02
Trp	− 3.68E–03	4.96E–03	135	− 3.17E–03	− 3.20E–03
Tyr	− 4.25E–03	3.59E–03	84.5	− 5.28E–03	− 9.82E–03
Val	− 1.45E–02	6.64E–03	45.8	− 1.51E–02	− 1.25E–02

The literature flux values were obtained from (1) Carinhas et al. (2013) and (2) Selvarasu et al. (2012). The fluxes units are in mmol/gDW/h for all metabolites and h^−1^ for the cell growth rate

Putting all constraints together results in a LP with mechanistic decision variables (metabolic fluxes, $v$) and empirical decision variables (PCs $\mathrm{Scores}$) stated as follows:

6 $$\underset{v, Scores}{\mathrm{minimize}}\{J={c}_{v}^{T}v+{c}_{\mathrm{Scores}}^{T}\mathrm{Scores}\}$$

Subject to (a) parametric (mechanistic) constraints:

$$0=\left[\begin{array}{cc}{S}_{\mathrm{int}},& 0\end{array}\right]\left[\begin{array}{c}v\\ \mathrm{Scores}\end{array}\right]$$

$${\mathrm{LB}}^{v}\le v\le {\mathrm{UB}}^{v}$$

(b) nonparametric (empirical) constraints:

$${\mu }_{\mathrm{vPCA}}-\mathrm{RF}\bullet {\sigma }_{\mathrm{vPCA}}\le \left[\begin{array}{cc}{S}_{\mathrm{ext}},& -{\sigma }_{\mathrm{vPCA}}\circ \mathrm{Coeff}\end{array}\right]\left[\begin{array}{c}v\\ \mathrm{Scores}\end{array}\right]\le {\mu }_{\mathrm{vPCA}}+\mathrm{RF}{\bullet \sigma }_{\mathrm{vPCA}}$$

$${\mathrm{LB}}^{\mathrm{Scores}}\le \mathrm{Scores}\le {\mathrm{UB}}^{\mathrm{Scores}}$$

(c) upper/lower bounds of exchange fluxes:

$${r}_{\mathrm{mean}}-k\sigma \le \left[\begin{array}{cc}{S}_{\mathrm{ext}},& 0\end{array}\right]\left[\begin{array}{c}v\\ \mathrm{Scores}\end{array}\right]\le {r}_{\mathrm{mean}}+k\sigma$$

The vectors ${c}_{v}$ and ${c}_{\mathrm{Scores}}$ (with the same dimension of $v$ and $\mathrm{Scores}$ respectively) are used to set the objective function (+ 1/− 1 coefficients are chosen to minimize/maximize a particular flux and/or score). Given the semiparametric nature of HybridFBA, a calibration step of the nonparametric constraints is always needed. The most important parameter is the number of PCs (NPC). The NPC corresponds to the number of scores that the LP optimizes and also to the number of columns of the $\mathrm{Coeff}$ matrix. The optimal NPC value should be chosen to maximize the predictive power of the HybridFBA (more to this in the results section). The other parameter is the relaxation factor (RF). The RF sets the maximum admissible absolute error between the mechanistic and empirical exchange flux solution, $\left|{\varepsilon }_{\mathrm{rexch}}\right|$:

7 $$\frac{\left|{\varepsilon }_{\mathrm{rexch}}\right|}{{\sigma }_{\mathrm{vPCA}}}\le RF$$

When RF = 0, Eq. (4) reduces to Eq. (3) thus the mechanistic and PCA constraints must exactly match each other. When RF = ∞, the PCA constraints have no effect in the LP, and HybridFBA becomes analogous to standard FBA. In practice, RF should be chosen as small as possible under the limit of a feasible LP.

This LP problem was solved by constrained linear programming using the dual-simplex algorithm with arbitrarily large number of iterations (MATLAB linprog function). The algorithm terminates when an optimal solution is reached. The linprog function also computes the Lagrange multipliers of all equality and inequality constraints at the optimal solution. The Lagrange multipliers were used for shadow price analysis [31]. The Lagrange multipliers display the sensitivity of the objective function to the constraints. The Lagrange multiplier of a given constraint may be interpreted as the increase in the objective function value when the constraint is relaxed by 1 unit. The MATLAB code of the HybridFBA method together with a toy example are provided in the supplementary material.

### Design of a cell growth feed

A feed composition and feed rate controller were designed from the HybridFBA fluxes and then validated in a 5 L reactor experiment. The design principle was to feed along time the computed substrates consumption by the HybridFBA method. More specifically, the following steady-state material balance was applied:

8a $$FC_F = r_{{\text{exch}}} X_V V$$

with $F$ the feed rate in mL/h, ${C}_{F}$ a vector of concentrations in the feed (mmol/mL), ${r}_{\mathrm{exch}}$ the consumption rates of substrates in mmol/(Mcell×h) (computed by HybridFBA), ${X}_{V}$ the viable cell count measured online in Mcell/mL and $V$ the culture volume measured online in mL. The concentrations of substrates were computed in relation to glucose, ${c}_{\mathrm{Glc}}$,

8b $${C}_{F}={c}_{\mathrm{Glc}}\frac{{r}_{\mathrm{exch}}}{{r}_{\mathrm{exch},\mathrm{ Glc}}}$$

The feeding rate controller is per mathematical equivalence given by the following equation.

8c $$F=\frac{{r}_{\mathrm{exch},\mathrm{ Glc}}}{{c}_{\mathrm{Glc}}}{X}_{V}V=a{X}_{V}V$$

A feed solution was formulated according to Eq. (8b). The reference Glc concentration was chosen such that all compounds are below 75% of the solubility limit. The feed controller [Eq. (8c)] requires the on-line measurement of ${X}_{V}$ (VCD in Mcell/mL obtained online by the Vi-cell counter) and $V$ ((estimated) culture volume, mL). It is a feedforward controller whereby the feed of nutrients reacts to the “perturbation” of higher/lower VCD inside the reactor.

## Results and discussion

### Experimental flux data set

**Fig. 2:**
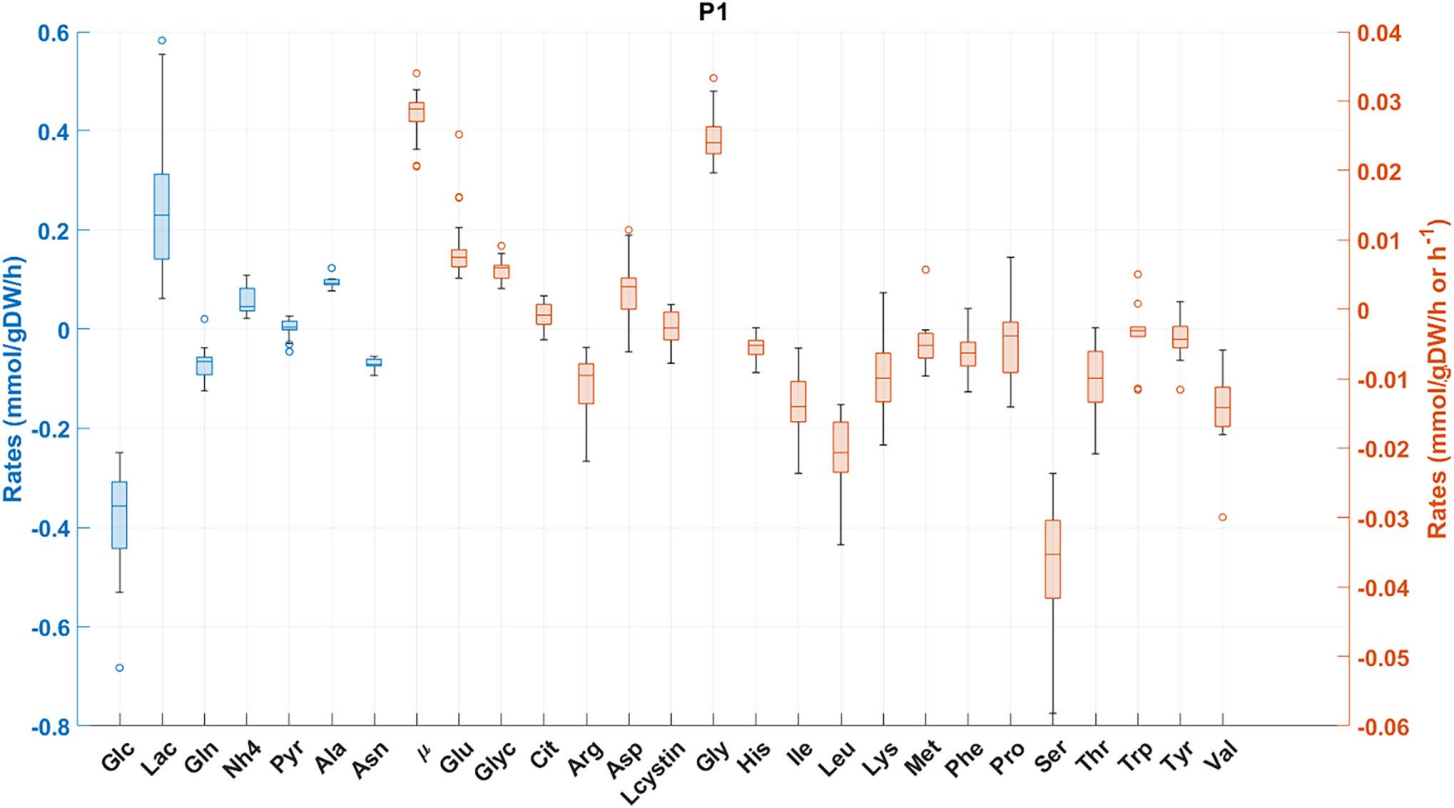
Box-plot of fluxes during exponential cell growth aggregated from 21 independent CHO cultivations. The bar represents the median. the box is the first and third quartile, and the whisker the minimum and maximum of the fluxes. Blue axis: fluxes with higher values. Orange axis: fluxes with lower values)

Experimental flux values of 27 extracellular species were estimated from time series data of 21 reactor experiments. Figure 2 shows the flux data dispersion among the 21 reactor experiments during exponential growth (corresponding approximately to the initial 70 h of cultivation). The mean flux values and respective standard deviations are compared with literature data in Table 1. The literature data [32, 33] refer to the exponential growth of CHO-K1SV and CHO-K1 cell lines respectively. The experimental fluxes obtained in this study are in range of literature data despite the different cell lines and cultivation protocols. The maximum specific growth rate attained was 0.0282 h^−1^ with a coefficient of variation (CV) of 11.9% (among the 21 reactor experiments), on par with literature data (Table 1). The measured fluxes reveal signs of a “healthy” cell growth, as there is accumulation of Gly, from the serine (Ser) metabolism, and Glyc, which is an indication of high (NADH/NAD +) redox state [34]. All amino acids but Ala, Aspartate (Asp), Gly and Glutamate (Glu) were consumed during exponential growth. The amino acids with highest consumption rates were Gln, Ser and Leu, with rate values comparable to literature data (Table 1).

The CHO cells used in this study consumed more Glc compared to the literature and, consequently, more Lac was produced (Table 1). Nevertheless, the ratio of Lac to Glc was significantly lower than 1, similar to other studies ([32, 33], Table 1). Alternative fates of Glc are the pentose phosphate pathway or intracellular Pyr accumulation. The latter can be secreted or used to produce Ala, or proceed to the tricarboxylic acid cycle (TCA). High glycolytic activity with low Lac to Glc ratio is coherent with Pyr release to the medium (observed in this study in opposition to literature data) and also with a higher Ala release rate to the medium (Table 1).

Gln was consumed concomitantly with Glu, NH4 and Asp accumulation. Glu is typically found in excess intracellularly due to its numerous sources, namely Ala, Gln, Lysine (Lys) and proline (Pro) [35]. The NH4 accumulation flux is comparable to other studies ([32, 33], Table 1), linked to amino acids catabolism and to direct degradation of Gln in the medium. Asp may accumulate from asparagine (Asn) via L-asparaginase [36] or via aspartate transaminase (AspTA), a reversible reaction that produces Asp and α-ketoglutarate (Keto) from Glu and OAA [37].

### Principal component analysis

**Fig. 3:**
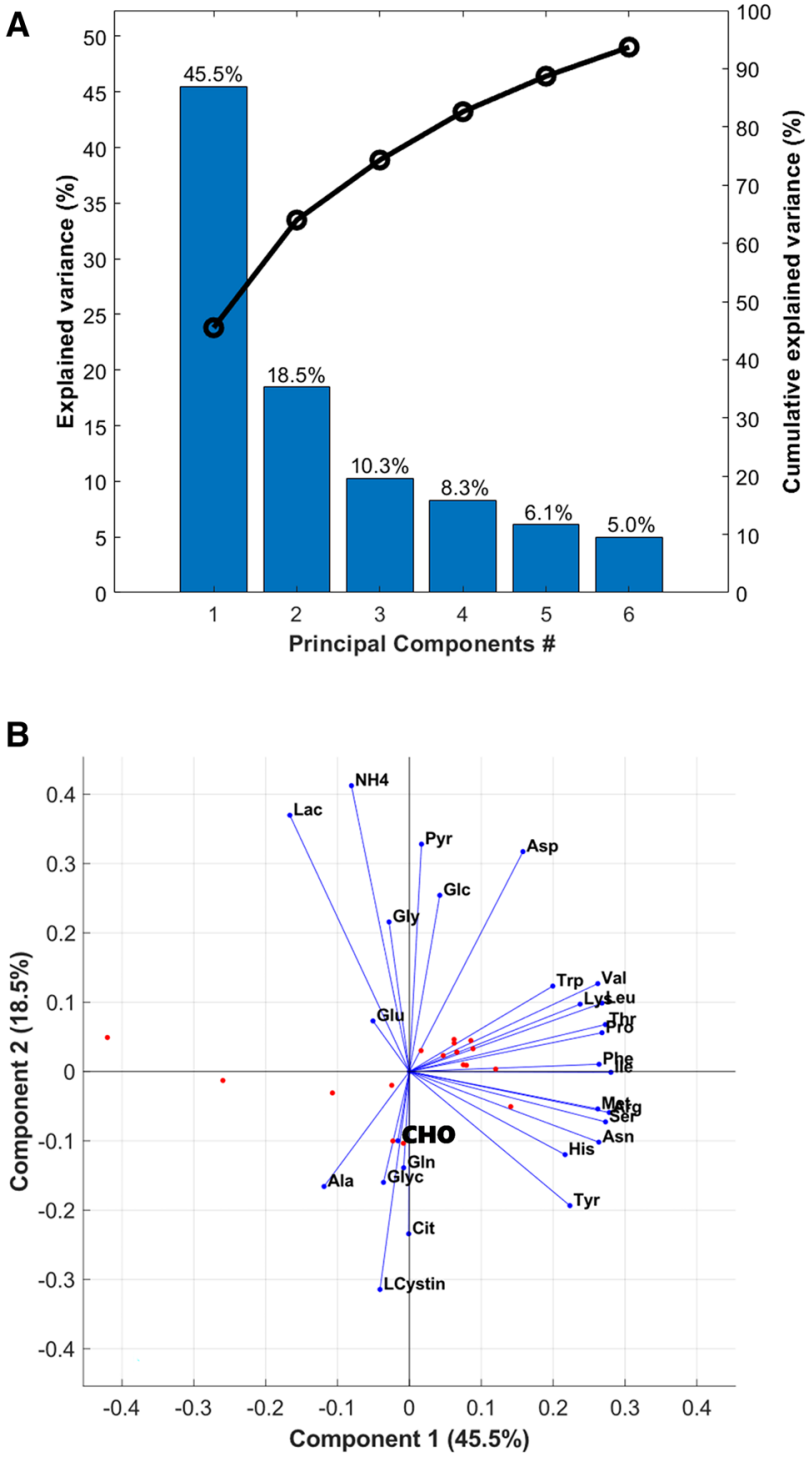
Explained variance captured by PCA of flux data obtained from 21 reactor experiments and 27 extracellular rates (process descriptors) during exponential growth. **A** Explained variance of flux data by each PC (left axis) and cumulative explained variance (right axis). **B** Biplot of Principal Component 2 (18.5% explained variance) over Principal Component 1 (45.5% explained variance). Red dots represent score values of the 21 reactor experiments. Blue vectors represent the coefficients of process descriptors

The estimated maximum specific growth rate has a CV of 11.9% among the 21 reactor experiments. The other fluxes show, however, a higher dispersion. The Glc uptake has a CV of 27.3%. Lac, Gln, Glu and NH4 have CVs close to 50%. The observed dispersion may be caused by experimental error or by multiple physical processes such as reactor operation variability, inoculum variability and different feed programs. Metabolic switches between by-product release or uptake may occur in CHO cells, for example for lactate [38], which may lead to large variations especially in cultivations with different cell growth conditions. The flux data set was subject to PCA for a better understanding of potential variation causes (Fig. 3). The 27 process descriptors (the measured rates) could be compressed to 6 orthogonal PCs with explained variance higher than 90%. The cumulative explained variance by PC 1-to-6 was 45.5, 64.0, 74.3, 82.6, 88.7 and 93.7% as shown in Fig. 3A. A significant part of fluxes variances is therefore dictated by metabolic mechanisms rather than by random error.

Figure 3B shows the biplot of PC-2 over PC-1 (the 2^nd^ column of the $\mathrm{Coeff}$ matrix is plotted against the 1^st^ column) evidencing strong correlations between groups of fluxes. It stands out that the coefficients of the cell growth rate are low in comparison to the other descriptors, indicating that the cell growth rate is less sensitive to process variation. Also, Gln and Glu have moderate contributions to data variance (low coefficients in matrix $\mathrm{Coeff}$). On the contrary, the Glc flux appears strongly correlated with Lac and NH4 along the direction of PC-2. The consumption of most amino acids appears highly correlated with each other (along the direction of PC-1). Some amino acids fluxes are positively correlated with Lac and NH4 fluxes, while others are negatively correlated. These observations are compatible with high and variable glycolytic activity with minor impact on the cell growth rate. It seems to be theoretically possible to modulate metabolism to minimize the accumulation of byproducts while maintaining exponential growth.

### Flux balance analysis

**Fig. 4:**
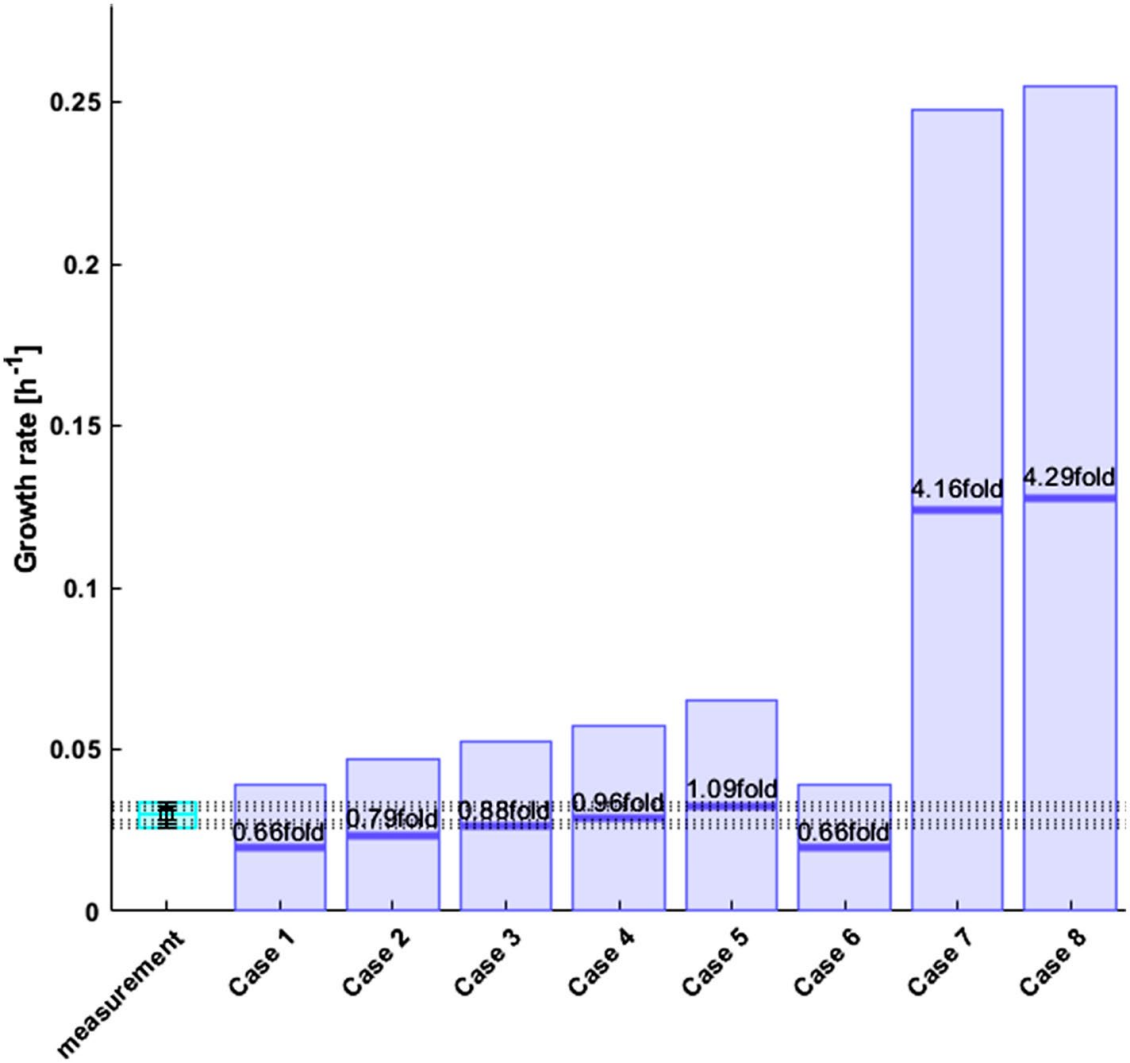
Predicted specific growth rate by standard FBA for 8 test case scenarios. The light blue bar represents the mean experimental value. The horizontal dashed lines represents the experimental + 1σ, + 2σ and + 3σ boundaries. The dark blue bars represent the computed specific growth rate interval by the model. The middle blue dash represents the predicted half-interval value **Case 1** All exchange fluxes except the specific growth rate were constrained to the $\pm 1\sigma$ domain. **Case 2** All exchange fluxes except the specific growth rate were constrained to the $\pm 2\sigma$ domain. **Case 3** All exchange fluxes except the specific growth rate were constrained to the $\pm 3\sigma$ domain. **Case 4** All exchange fluxes except the specific growth rate were constrained to the $\pm 4\sigma$ domain. **Case 5** All exchange fluxes except the specific growth rate were constrained to the $\pm 6\sigma$ domain. **Case 6** The same as case 1 for substrates only. All substrates are constrained to the $\pm 1\sigma$ domain. The products have unlimited lower/upper bounds. **Case 7** Only Glc and Gln are constrained to the mean $\pm 1\sigma$ domain. All other compounds had unlimited bounds. **Case 8** Only Glc and Gln are fixed to the mean $\pm 3\sigma$ domain. All other compounds had unlimited bounds. The sum of squares of residuals (difference between the experimental mean and predicted half-interval value) was 1.68

Standard FBA was performed assuming metabolic optimality towards the production of biomass during exponential growth [12, 16, 39, 40]. More specifically, HybridFBA was applied to maximize the specific growth rate with NPC = 0 for different test case scenarios. Making NPC = 0 removes the inequality constraints [Eq. (4)] and transforms HybridFBA into a standard FBA. In each test case scenario, the specific cell growth rate was both maximized and minimized to obtain a minmax solution interval, to better display the sensitivity of the FBA solution within the constraints domain. The overall results are shown in Fig. 4.

In test case 1, FBA was applied with all exchange fluxes but the specific growth rate constrained to the $\pm 1\sigma$ domain (Eq. (5) with $k$=1). In other words, no boundaries were set for the specific growth rate whereas all other measured exchange fluxes were free to move within the ${r}_{\mathrm{mean}}-1\sigma \le {r}_{\mathrm{exch}}$ ${\le r}_{\mathrm{mean}}+1\sigma$ space (the ${r}_{\mathrm{mean}}$ and $\sigma$ are given in Table 1). This amounts to 26 inequality constraints defined by exchange flux measurements. This analysis resulted in the specific growth rate interval of [0, 0.0393] h^−1^ with mean value of 0.0196 h^−1^. This interval comprehends the measured value but the predicted mean is 70% lower than the measured mean (0.0282 ± 0.0034 h^−1^).

Test cases 2–5 are similar to test case 1 except that the design interval (Eq. (5)) was progressively enlarged from the $\pm 1\sigma$ domain (*k* = 1 in test case 1) up to the $\pm 6\sigma$ (*k* = 6 in test case 5). As a result, the specific growth rate minmax interval also increased to [0, 0.0649] h^−1^. In test case 6, only the substrates were constrained to the $\pm 1\sigma$ domain. All products including biomass were unbounded. The resulting specific growth rate interval was the same as in test case 1 (where only biomass was unbounded).

Finally, in test case 7 and 8, only Glc and Gln were bounded in the design intervals of $\pm 1\sigma$ and $\pm 3\sigma$ respectively. This very flexible scenario resulted in a substantial enlargement of the FBA specific growth rate minmax interval [0, 0.125] h^−1^ (8.6 fold increase in relation to the mean measured value).

All in all, these results show that the FBA solution interval always contains the measured mean value. The minmax solution tends to be much wider than the measurement variance. In some cases (test case 7 and 8) there is a significant offset between the predicted and measured mean values. The sum of squared residuals was 1.68 for the 8 test cases.

### Hybrid semi-parametric flux balance analysis

The HybridFBA method was applied for the same test cases 1–8 with the inclusion of the PCA constraints by making NPC > 0. Since the PCA was applied to the measured extracellular rates, Eq. (4) adds 27 inequality constraints. On the other hand, HybridFBA has more decision variables than standard FBA. It optimizes the flux values of all GEM reactions (as in standard FBA) and additionally the score values associated to the *NPC* PCs (*NPC* is a calibration parameter). The number of additional constraints is however higher than the number of additional decision variables. As such, HybridFBA has (*27*–*NPC*) less degrees of freedom than standard FBA. This reduction is always effective as long as the PCA compresses the measured rate data into a lower dimension PCs space (e.g., *NPC* ≪27*)*.

**Fig. 5:**
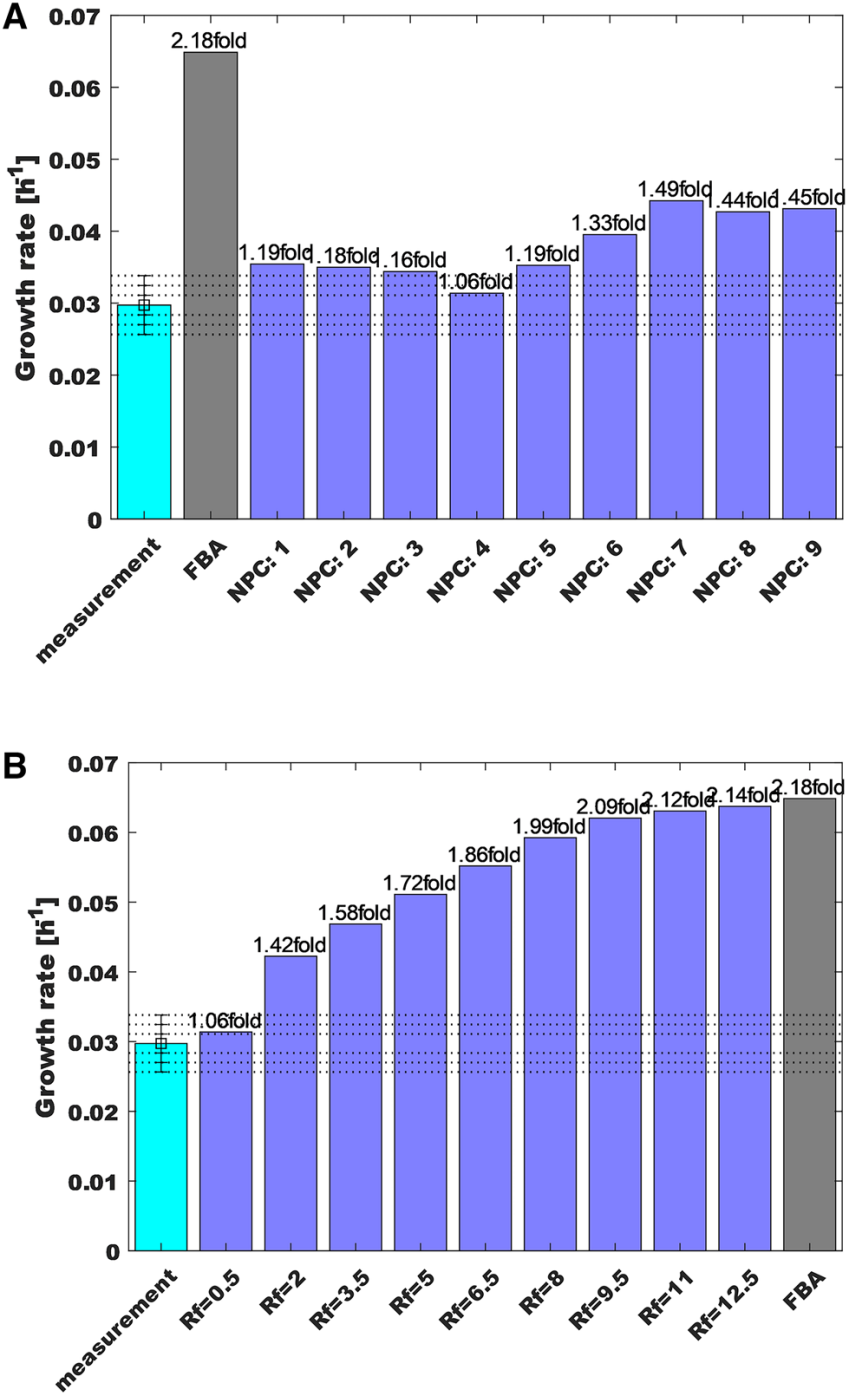
Calibration of the HybridFBA method. **A **Predicted maximum specific growth rate as function of the number of principal components in the hybrid model [Eq. (4)] for a relaxation factor RF = 0.5. **B **Predicted maximum specific growth rate as function of the relaxation factor value for number of principal components NPC = 4

The HybridFBA method was firstly calibrated using the test case 5 as base scenario because it has the widest $\pm 6\sigma$ domain. Figure 5A shows the predicted maximum specific growth rate as function of the *NPC* for arbitrarily small relaxation factor (RF = 0.5). The first column (light blue column) is the reference (experimental) mean value. The second column (NPC = 0) is the previously discussed standard FBA solution for test case 5, which predicted a 2.18 fold increase in relation to the experimental mean value. All other solutions were obtained with *NPC* between 1 and 10. Indeed, the predicted specific growth rate depends on the number of PCs chosen. The closest prediction to the measured specific growth rate was obtained with NPC = 4. Figure 5B shows the effect of RF on the HybridFBA solution when fixing NPC = 4. As expected, when the RF increases the PCA constraints [Eq. (4)] are relaxed with the HybridFBA solution eventually converging to the standard FBA solution.

**Fig. 6:**
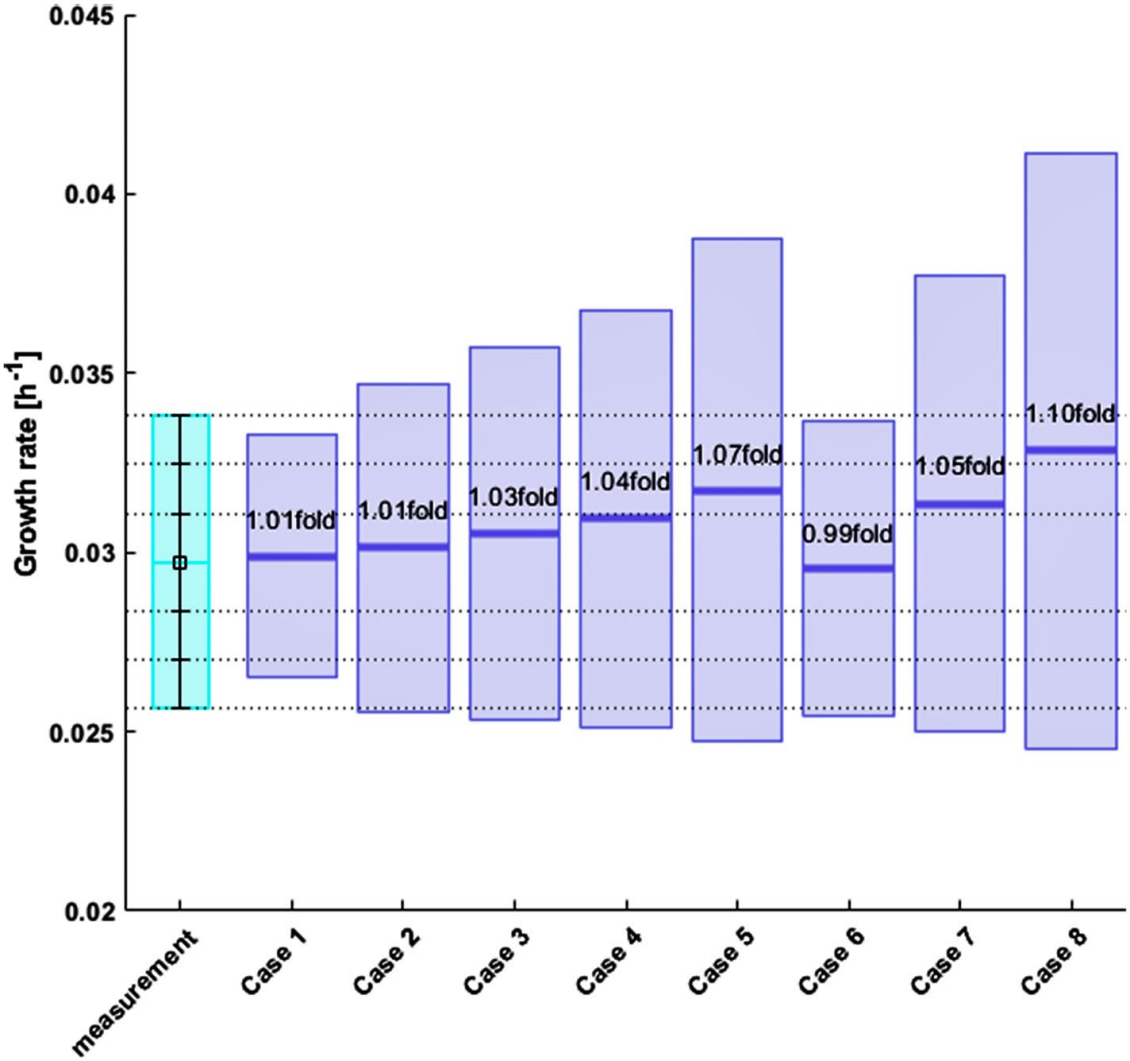
Predicted specific growth rate by HybridFBA with number of principal components NPC = 4 and relaxation factor RF = 1.0 for 8 test case scenarios. The light blue bar represents the mean experimental value. The horizontal dashed lines represents the experimental + 1σ, + 2σ and + 3σ boundaries. The dark blue bars represent the computed specific growth rate interval by the model. The middle blue dash represents the predicted half-interval value **Case 1** All exchange fluxes except the specific growth rate were constrained to the $\pm 1\sigma$ domain. **Case 2** All exchange fluxes except the specific growth rate were constrained to the $\pm 2\sigma$ domain. **Case 3** All exchange fluxes except the specific growth rate were constrained to the $\pm 3\sigma$ domain. **Case 4** All exchange fluxes except the specific growth rate were constrained to the $\pm 4\sigma$ domain. **Case 5** All exchange fluxes except the specific growth rate were constrained to the $\pm 6\sigma$ domain. **Case 6** The same as case 1 for substrates only. All substrates are constrained to the $\pm 1\sigma$ domain. The products have unlimited lower/upper bounds. **Case 7** Only Glc and Gln are constrained to the mean $\pm 1\sigma$ domain. All other compounds had unlimited bounds. **Case 8** Only Glc and Gln are fixed to the mean $\pm 3\sigma$ domain. All other compounds had unlimited bounds. The sum of squares of residuals (difference between the experimental mean and predicted half-interval value) was 0.0016

After calibration, HybridFBA with NPC = 4 and RF = 1.0 was applied to minimize/maximize the specific growth rate under the same constraint scenarios previously adopted for standard FBA (It should be noted that RF = 1.0 was the lowest value that complied with the constraints of all test cases 1–8). The overall results are shown in Fig. 6. The specific growth rate interval contains the experimental mean value in every test case. Contrary to standard FBA, the predicted specific growth rate half interval is within the ± 2σ experimental bounds in all test cases. HybridFBA showed a significant improvement particularly for the more flexible test cases 7 and 8 (only Glc and Gln were constrained whereas all other compounds were unbounded). While HybridFBA slightly overpredicted the specific growth rate (1.05 fold and 1.10 fold of the mean) standard FBA showed a four fold plus off-set in relation to the experimental mean. The HybridFBA sum of squared residuals (SSR) was 0.0016 for the 8 test cases, which is 3 orders of magnitude lower than the SSR obtained by standard FBA (1.68). The HybridFBA solution interval is also much narrower when compared to the FBA solution interval. The standard FBA always found a feasible null growth solution in all scenarios (when the objective function was set to minimize the specific growth rate). On the contrary, the HybridFBA always found a positive minimal growth solution with a minmax interval close to symmetry in relation to the mean experimental value.

**Fig. 7:**
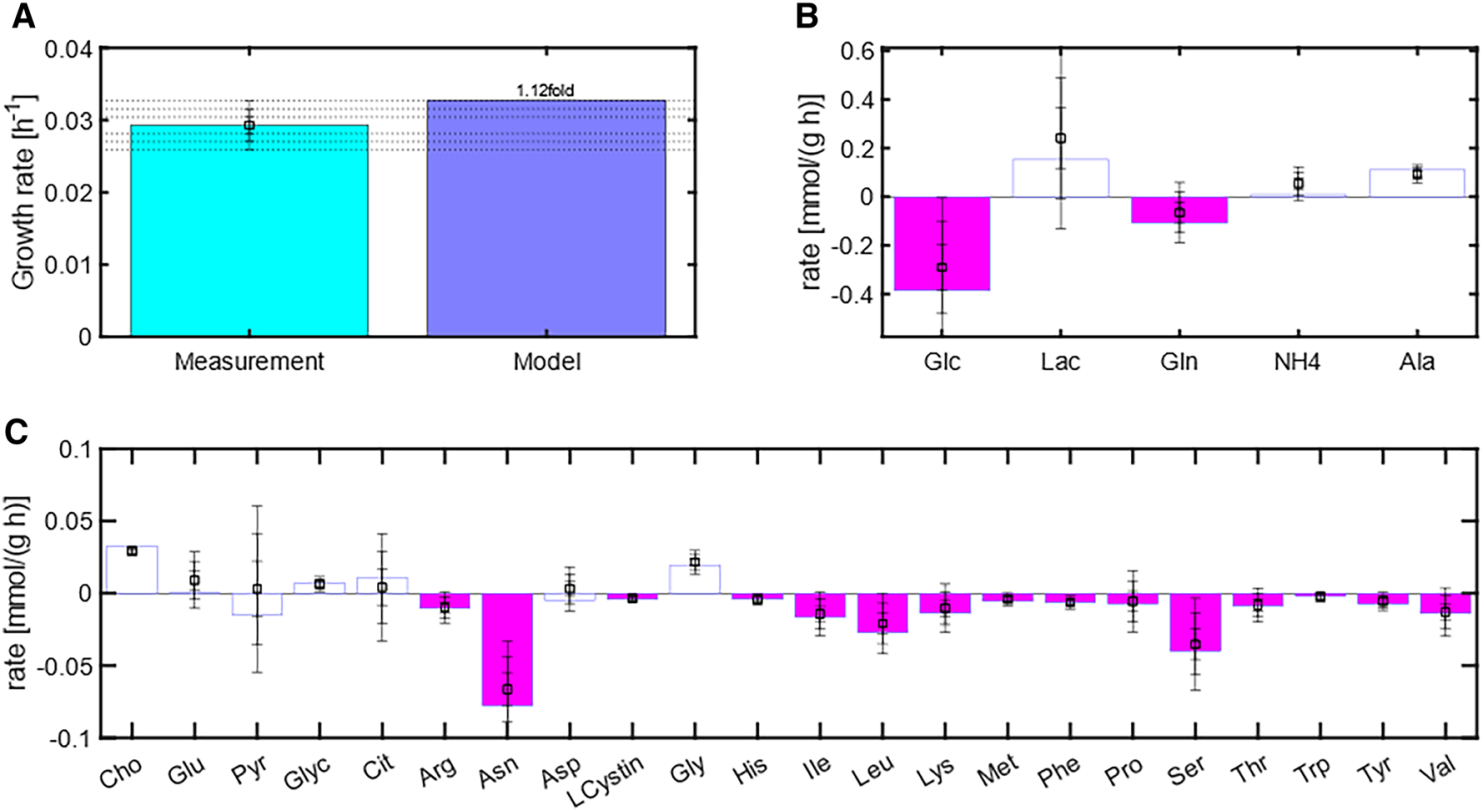
Comparison between experimentally measured fluxes and HybridFBA predictions for test case 6 (All substrates were constrained to the ± 1 $\sigma$ domain whereas all products had unlimited lower/upper bounds). **A** Specific growth rate (objective). Light blue bar is the measured specific growth rate. Dark blue is the respective model prediction. **B, C** Predicted (bars) versus measured mean (square) with 1$\sigma$, 2$\sigma$ and 3$\sigma$ experimental bounds. Pink bars refer to the prediction of the substrate rates that were constrained to mean ± 1$\sigma$. The open blue bars represent the prediction of the unconstrained product fluxes

Figure 7 shows in more detail the specific growth rate maximization solution for test case 6 (this test case will the basis for the design of an optimal feed in the next section). In test case 6, only substrates were constrained to the ± 1σ domain whereas the rates of products (specific growth rate) and byproducts (Lac, NH4, Ala, Glu, Pyr, Glyc, Asp and Gly) were unbounded. The HybridFBA predicted a maximum specific growth rate, which is 1.12fold of the mean experimental value. The pink bars show the calculated substrate fluxes. With few exceptions, the substrate fluxes converged very close to the lower bound (${r}_{\mathrm{mean}}$− 1σ) (pink bars in Fig. 7), which is consistent with the biomass production maximization objective. Noteworthy, the fluxes of the unconstrained products (open blue bars in Fig. 7) are all within the experimental domain. For example, the predicted accumulation rates of Lac (0.234 mmol/gDW/h), NH4 (0.082 mmol/gDW/h) and Ala (0.105 mmol/gDW/h) are very close to the respective experimental values (0.208 mmol/gDW/h, 0.050 mmol/gDW/h and 0.094 mmol/gDW/h respectively). This is in deep contrast with the standard FBA solution, which predicted unrealistic byproduct fluxes (for example, Lac, 2.167 mmol/gDW/h, NH4, 1.993 mmol/gDW/h, Ala, − 1.883 mmol/gDW/h) far off the experimental bounds.

### Design of a metabolically efficient cell growth feed (OptCHO)

Given the relative success of HybridFBA to predict (by) products fluxes from substrates fluxes, a culture medium feed was designed in silico. The objective was to extend the cell growth phase targeting a higher VCD at the time of induction with minimal byproducts accumulation. The HybridFBA was set to minimize the sum of byproducts secretion rates (Lac, NH4, Ala, Glu, Pyr and Asp; Gly and Glyc were excluded as they are associated with a healthy growth phenotype [34]) whereas the substrates fluxes were constrained to the ± 3σ domain (Eq. (5) with *k* = 3; ${r}_{\mathrm{mean}}$ and *σ* given in Table 1). Additionally, the specific growth was fixed to the target 0.0282 h^−1^ (mean experimental value ± 5% variation to introduce some flexibility in the optimization). Table 2 summarizes the design results obtained by HybridFBA. The final objective function value was ${J}_{\mathrm{OptCHO}}=- 0.013\frac{\mathrm{mmol}}{\mathrm{gDW}.\mathrm{h}}$. A generic decrease in substrates fluxes (with exception of Gln) is forecasted concomitantly with a generic decrease of byproducts accumulation. It should be noted that Lac, Pyr, Glu and Asp inverted their role as byproducts in the reference scenario to become substrates in OptCHO. The scores of principal components 1 and 2 calculated by HybridFBA were − 0.78 and − 4.71 respectively, thus scoring OptCHO in the left/lower quadrant in the biplot of Fig. 3B. This is coherent with the maximization of the growth rate simultaneously with the minimization of substrates and byproducts. Table 2 also shows the Lagrange multipliers at the optimal solution for the substrates and products exchange flux constraints. The Lagrange multipliers display the sensitivity of the objective function to the constraints and are interpreted as shadow prices in LP [31]. These data suggest that relaxing the boundaries of the cell growth rate (µ) and of the exchange fluxes of Phenylalanine (Phe), Tyrosine (Tyr), Arginine (Arg), NH4 and Glu could further improve the objective function value. We have further investigated the influence of biomass composition in the OptCHO solution (details given the supplementary material). This analysis showed that biomass composition uncertainty does not significantly affect the estimation of the specific growth rate and that it mostly impacts the low range flux values.

**Table 2:**
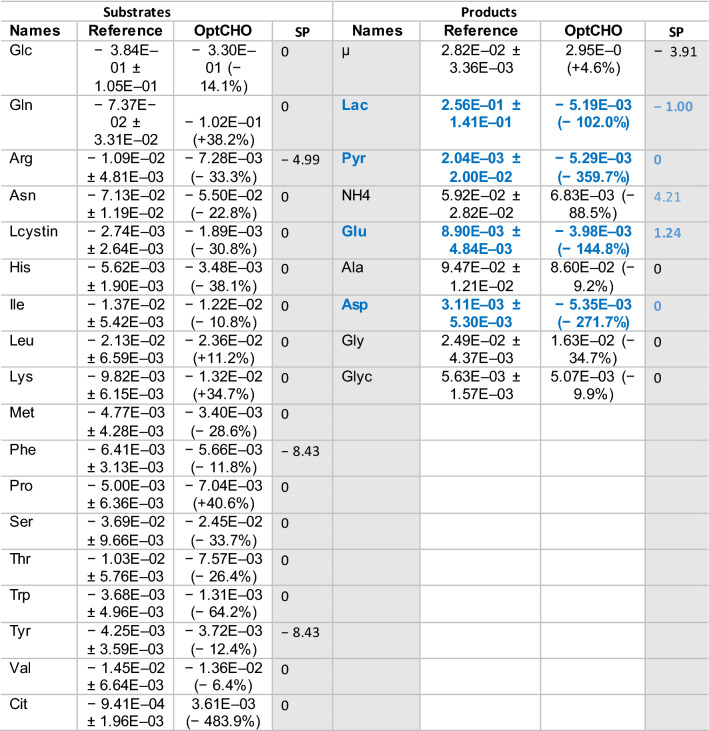
OptCHO fluxes designed by HybridFBA in comparison to the reference fluxes (experimental data and respective standard deviation), along with the shadow prices (SP) of the OptCHO solution

In blue the metabolites that are byproducts in the reference condition and substrates in the OptCHO design. The fluxes units are in mmol/gDW/h for all metabolites and h^−1^ for the cell growth rate. The percentual variation in parenthesis refers to the OptCHO compared to the reference fluxes

To gain a better understanding of the metabolic patterns underlying OptCHO, the activities of the GEM subsystems were analyzed relatively to the reference condition (Table 2). For this analysis, FVA was applied for evaluating alternative optima that can still satisfy the OptCHO constraints within a ± 5% objective function tolerance. More specifically, the minimum and maximum flux range of each reaction were calculated with the previously described HybridFBA method using the additional constraint:

9 $${0.95J}_{\mathrm{OptCHO}}{\le c}_{v}^{T}v+{c}_{\mathrm{Scores}}^{T}\mathrm{Scores}\le {1.05J}_{\mathrm{OptCHO}}$$

**Fig. 8:**
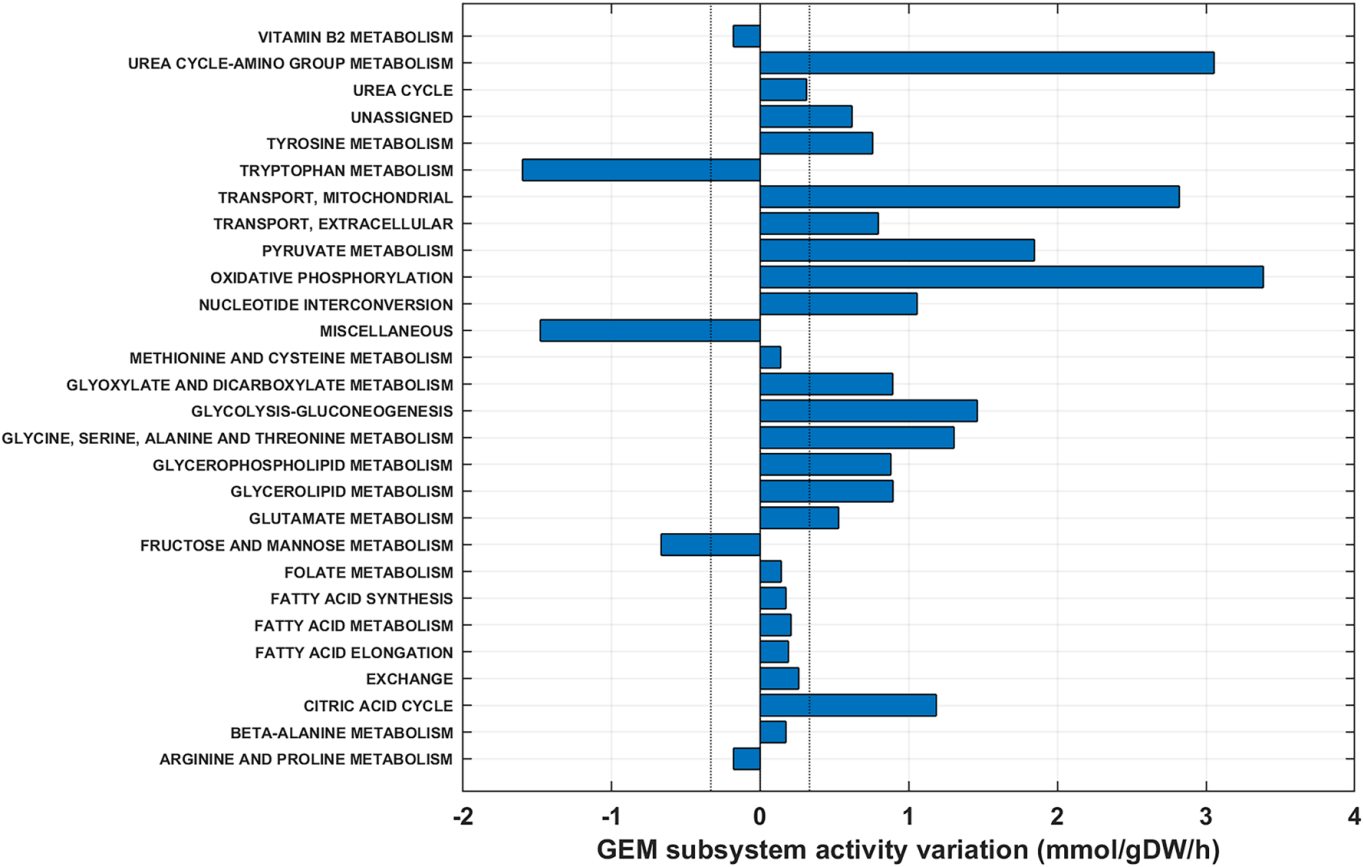
GEM subsystems activity variation of the OptCHO flux solution (half-interval flux solution obtained by FVA) in relation to the reference condition. Blue bars represent the subsystem activity variation in mmol/gDW × h in relation to the reference condition. The subsystem activity is computed as the sum of the absolute value of in/out fluxes to the subsystem. A value of 0 means that OptCHO activity is equal to the reference condition activity. A value ≫ 1 means that the subsystem is up-regulated in relation to the reference condition. A value ≪1 means that the subsystem is down-regulated in relation to the reference condition

Figure 8 shows the change of GEM subsystems activity of the FVA half interval fluxes relatively to the reference condition. The TCA activity in OptCHO is significantly increased mainly fueled by the higher Gln and Asp consumption rates (e.g., [41]) and to a less extent by Pyr uptake (instead of secretion). Increased TCA activity indicates a more efficient metabolism [42], which is consistent with the increase of oxidative phosphorylation, nucleotide interconversion, and urea cycle/amino group metabolism subsystems. Lac consumption indicates that the Warburg effect is avoided (Glc mainly used for Lac production, [43]), suggesting a more efficient metabolism. The OptCHO solution relatively increased glycolysis/gluconeogenesis activity despite the significant reduction in Lac secretion. Higher glycolytic rates with lower Glc consumption are explained by TCA intermediates recycling in the glycolysis. The increase of the mitochondrial transport activity further sustains the exchange of intermediates between glycolysis and other subsystems such as TCA. This exchange subsystem also contributes to the increase in pyruvate metabolism (transport to mitochondria and conversion of pyruvate to TCA intermediates). The increase in Pyr metabolism is also due to the Pyr and Lac uptake (flux inversions in relation to the reference).

### Example of process implementation

**Fig. 9:**
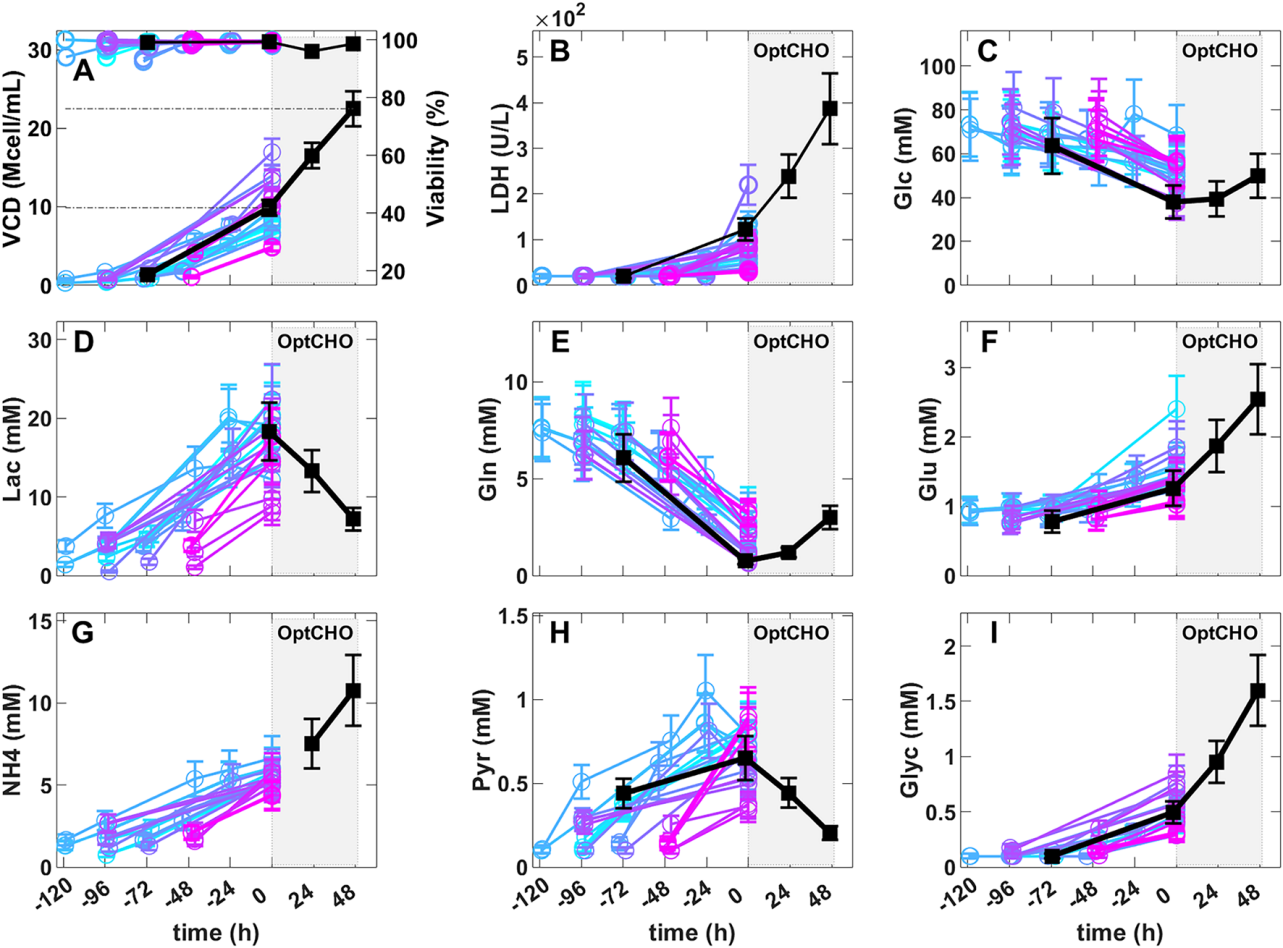
Validation of the OptCHO feed in a 5 L reactor experiment. The colored lines and symbols represent the concentrations ± SD for the batch phase of 21 historical experiments. The black line and symbols refer to the design experiment where OptCHO feed was applied. Time 0 h corresponds to the onset of the OptCHO feeding in the design experiment and to the induction in the 21 historical experiments. The gray shadow marks the time window (48 h) for the OptCHO controller operation. **A**, **B, C, D, E, F**, **G**, **H, I** Measured concentrations of VCC, LDH, Glc, Lac, Gln, Glu, NH4, Pyr, and Glyc over process time

Here we illustrate how the hybrid FBA exchange flux solutions can be quasi-automatically translated into culture media feeds. A feed composition and feed rate controller were implemented in a 5 L reactor experiment based on the OptCHO exchange fluxes (Table 2). A concentrated feed solution was formulated obeying to the mol/mol stoichiometric ratios of the OptCHO solution (see methods section). A feedforward controller was designed that adjusts the feeding rate based on the VCD online measurement by the Vi-Cell counter (see methods section). Figure 9 shows the dynamics of the OptCHO experiment until the temperature-shift induction (black line and symbols). Figure 9 also shows the dynamics of the 21 historical reactor experiments until the induction point (colored lines and symbols). The 21 historical experiments were inoculated with different cell concentrations and were induced at different time points. Thus the resulting cell growth and metabolic footprint dynamics are very diverse and difficult to compare. As for the OptCHO experiment, the dynamic profiles changed considerably before and after the controller started operation. The OptCHO feed controller automatically started at a VCD of 9.87 Mcell/ml. The VCD increased to 22.48 Mcell/ml after 48 h operation (gray shadow of Fig. 9A). The CHO culture tolerated well the in silico OptCHO feed. As expected, the LDH essay suggests an increase of the number of dead cells with the increase of VCD (Fig. 9B). The LDH essay does not show an increase in the cell death rate during OptCHO operation. Moreover, a high cell viability > 98% was maintained during OptCHO operation (Fig. 9A). The average growth rate was 0.015 h^−1^ thus lower than the design. This may be partially explained by the feedforward control strategy, which does not necessarily enforce exponential growth. Moreover, OptCHO predicted Lac, Pyr, Glu and Asp as substrates (rather than byproducts), but they were not included in the feed. The Glc concentration was kept within the 40–60 mM interval (Fig. 9C). Lac decayed from 18.32 to 7.22 mM in conformity with the design (Fig. 9D). The Gln concentration is kept low within the 0–4 mM interval (Fig. 9E). There is a buildup of Glu (Fig. 9F) and NH4 (Fig. 9G) partially in conflict with the design. The NH4/biomass yield was however lower during OptCHO operation (0.42 micromol/Mcell) than in the preceding exponential growth phase (0.56 micromol/Mcell). This is qualitatively in agreement with the design since a reduction in the NH4 accumulation was predicted but not its inversion. Pyr was consumed during OptCHO operation in accordance with the design. The buildup of Glyc (and Gly) is associated with a healthy growth phenotype [34] and was not part of the objective function. The byproducts (Lac + Glu + NH4 + Pyr) yield was + 2.54 micromol/Mcell prior to OptCHO and then inverted to − 0.40 micomol/Mcell during OptCHO operation. These data suggests that the OptCHO partially succeeded to expand VCD concomitantly to the decrease of total byproducts accumulation. All in all, these results suggest that hybrid FBA exchange flux solutions can be translated into feasible culture media feeds, following a quasi-automatic standard procedure of process implementation.

## Conclusions

This study developed the concept of hybrid semi-parametric flux balance analysis with proof-of-principle the modeling and optimization of a CHO cultivation. Firstly, experimental fluxes were collected from 21 reactor experiments. The experimental fluxes portrayed a healthy cell growth phenotype with marked glycolytic activity and significant byproducts build-up during exponential growth. The flux data were analyzed by PCA and it was concluded that more than 90% of data variance could be explained by 6 PCs, evidencing strong correlations between measured fluxes. A hybrid semi-parametric flux balance analysis method (HybridFBA) was then developed that combines a reduced genome-scale model (GEM) with flux correlation constraints deduced from PCA. It was hypothesized that PCA derived constraints reflect the cellular regulatory mechanisms that control the uptake of nutrients and that the inclusion of this information could significantly increase the predictive power of standard FBA. This was confirmed in several specific growth rate prediction scenarios, showing that HybridFBA always predicted much closer to the experimental value than standard FBA. These results do not disqualify standard FBA as valid a design method as FBA can be further improved through the inclusion of additional constraints regarding enzyme kinetics, thermodynamics and/or regulatory processes, if such knowledge is available. The key message is that the inclusion of additional empirical constraints in a hybrid construct is likely to further improve the predictive power. Using this novel tool, a cell growth feed was designed in silico and tested in a lab experiment, showing that the viable cell count could be increase from 9.87 to 22.48 Mcell/ml with lower byproducts build-up with exception of Glu, which contradicted the design. One key advantage of the *HybridFBA* is the ability to learn from experience. While the GEM is a fixed part of the model, the PCA (and the hybrid ensemble per inherency) will improve with each new cultivation performed. This is aligned with the machine learning philosophy with the advantage of better interpretability through the GEM component. Lastly, the *HybridFBA* could be extended to the post-induction phase of different molecules. Other PCA constraints could be added to the GEM representing the unknown regulatory processes that control the assembly of the target molecule. In theory, with enough validation cycles across different molecules, the extended *HybridFBA* could be applied as a tool to design ab initio custom feeds for every new molecule.

## Supplementary Information

Below is the link to the electronic supplementary material.

Supplementary file1 (DOC 1240 KB)

Supplementary file2 (ZIP 5 KB)
