# Supplementary material for: Genome-scale modeling of Chinese hamster ovary cells by hybrid semi-parametric flux balance analysis
Source: Bioprocess Biosyst Eng. 2022 Oct 16;45(11):1889–904. doi: 10.1007/s00449-022-02795-9 (PMC9616788; doi:10.1007/s00449-022-02795-9)
Supplement: Supplementary file 1 — Supplementary file1 (DOC 1240 KB) [file 449_2022_2795_MOESM1_ESM.doc]

**SUPPLEMENTARY MATERIAL**

***Genome-Scale Modelling of Chinese Hamster Ovary Cells by Hybrid Semi-Parametric Flux Balance Analysis***

João R. C. Ramos^1^, Gil P. Oliveira^1^, Patrick Dumas^2^, Rui Oliveira^1^,*

*^1^LAQV REQUIMTE, Department of Chemistry, NOVA School of Science and Technology, NOVA University Lisbon, Campus Caparica, P-2829-516 Caparica, Portugal*

*^2^GLAXOSMITHKLINE BIOLOGICALS SA, 89, rue de l'Institut, 1330 Rixensart, Belgium*

^*^Corresponding author

Tel.: +351 212 948 356

Email: [rmo@fct.unl.pt](mailto:rmo@fct.unl.pt)

## ***Determination of extracellular fluxes***

Each reactor experiment was divided in 3 (quasi)steady-state phases: *P1*-Exponential cell growth (approximately 0-70 h), *P2*-Early stationary (approximately 190-300 h), *P3*-Late stationary (approximately 310-415 h). The time windows slightly varied from experiment to experiment and were adjusted accordingly. Steady-state extracellular reaction rates were estimated for each phase. The reaction rate of extracellular species *i*,
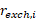
 with units mmol/(Mcell×h), was estimated from the following material balance equations:


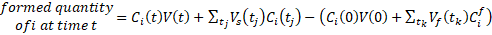

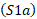


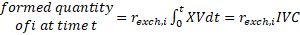
,
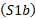


with
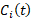
 the concentration of species
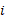
 (mmol/L) at time
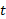
 (h),
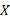
 the VCD (in units of Mcell/L),
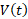
 the cultivation volume (L) at time
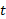
,
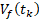
 the volume of feed (L) at feeding event
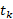
 (h),
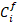
 the concentration of species
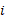
 in the feed (mmol/L),
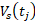
 the volume of sample (L) at sample event
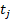
 (h). For the case of biomass, the quantity was defined in units of *Mcell* instead of *mmol*. Eq. 1a represents the formed quantity (due to reaction) of species
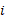
 at time t, which must balance the term
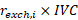
 (time Integral of VCD) in steady-state (Eq. 1b).

Due to sparse measurements in some experiments, Monte Carlo simulation was applied to generate additional data points. The formed quantity of species
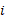
 was fitted against the IVC by splines filtering. Afterwards, 10 additional points were generated between samples by spline interpolation. Gaussian noise was added with standard deviation equal to that of the concentration multiplied by the culture volume at the respective time point.

The estimation of
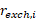
 then proceeded by robust linear regression of the formed quantity (measured and simulated by Monte Carlo simulation) against the IVC. The slope corresponded to the estimated reaction rate value,
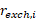
 . The standard deviation of slope, multiplied by the t-student (with 97.5% half-interval confidence and number of degrees of freedom equal to the number of time points – 1), provided the 95% confidence bounds of rate estimates,
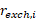
, for each phase of each experiment individually.

Glutamine (Gln) is chemically unstable decomposing with first-order kinetics into pyrrolidone carboxylic acid and ammonium (NH4) [1, 2]. The total amount of Gln that decomposes, resulting in an equimolar quantity of Nh4, was calculated as


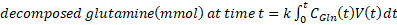

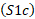


with
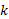
= 0.0016 h^-1^ [1]. As such, the balances of Gln and NH4 in Eq. (S1a) were modified by adding or subtracting to the formed quantity of Gln and NH4 respectively.

The biomass composition (and reaction) of the original CHO-K1 GEM [3] was adopted in this study (biomass composition was not measured in this study). The CHO cell dry mass during exponential growth was assumed 216.1 pg/cell. Viable cells were measured in units of Mcell/mL. The conversion factor from Mcell/mL to gDW/L was 0.2161. We analyze the sensitivity OptCHO solution to biomass composition below.

## **CHO-K1 GEM reduction**

The consensus CHO-K1 GEM [3], accessible in www.chogenome.org, was adopted in this study. This model contains 2773 metabolites, 4723 reactions and 2603 degrees of freedom. A reduction was performed based on previously published methodologies [4, 5]. More specifically, an 8 steps reduction procedure was applied as follows:

*Step 1*: The exchange reactions of species not present in the proprietary medium (with exception of the subset of free metabolites O_2_, CO_2_, H^+^, Cl^-^, Na^+^, K^+^, PP_i_, P_i_, and H_2_O) were removed from the model.

*Steps 2:* Redundancies in the exchange reactions of extracellular species were removed. Many compounds in the original CHO-K1 GEM have more than 30 exchange reactions. As many as possible of these reactions were eliminated but ensuring consistency with measured fluxes (mean ± 3σ) using FBA calculations.

*Step 3:* Metabolic sinks and bang-bang reactions were removed. Sinks are the synthesis of a component from an empty species or the consumption of a compound without any product formation. Bang-bang reactions are reactions that when maximized or minimized converge to the upper and lower bound limits respectively without affecting the extracellular fluxes.

*Step 4:* Thermodynamically infeasible loops were removed. A Loop is a result of cyclic reactions in which the fluxes do not add up to zero as described by Schellenberger et. al. [5]. These loops were identified using the constraint-based reconstruction and analysis (COBRA) framework in MATLAB ([6]; opencobra.github.io).

*Step 5:* Reactions leading to limitless or above expected values of ATP production were removed. Limitless ATP production leads to high ATP consumption for maintenance (R_DM_atp_c). The reactions that contribute to the high maintenance rate were identified by FBA and then removed.

*Step 6:* Reactions consuming ATP in a cyclic fashion without any metabolic purpose were removed. After this reduction, any overflow of ATP formation is directed for the maintenance reaction (R_DM_atp_c). These reactions are not essential and are easily discriminated by their null flux when applying FBA to maximize ATP production or to maximize biomass formation.

*Step 7:* Metabolic reactions were removed to ensure that the formation of ATP and its main precursors (NADH, FADH_2_) results from expected pathways, namely ATP synthase and key substrate-level phosphorylation reactions. This step ensures that large quantities of ATP and its main precursors are not produced by unexpected metabolic processes. It also limits the secretion of unexpected compounds to the medium and prevents the transport of ions against the membrane sodium gradient [4].

*Step 8:* In the final polishing of the reduced model, search and removal of bang-bang reactions was repeated. Finally, FVA was used to identify inactive reactions (reactions with flux <1e-15 in all scenarios), which were then removed.

In total 3935 intracellular reactions were eliminated in these steps. This process resulted in a reduced GEM, which is medium specific, containing 686 metabolites, 788 reactions and 210 degrees of freedom.

## **Influence of biomass composition**

We have investigated the influence of biomass composition in the OptCHO solution. Széliová et al. [2] reported variations in total protein content between 46-66% (w/w) in different cell lines and culture conditions. The relative amino acids composition was however almost invariant (up to 1.6% mol/mol variation only). The total lipids content varied slightly between 11.1-15.6% (w/w). The relative composition of some lipids varied up to 13.6% (mol/mol). Based on this information we performed a sensitivity analysis of the OptCHO solution to the biomass composition. The OptCHO was repeated 100 times with random modifications of selected biomass reaction coefficients. The total protein content was modified between 0-20% mol/gDW but keeping the relative amino acids composition constant. As for the lipids, modifications were limited to phosphatidylcholine, sphingomyelin, phosphatidylethanolamine and cholesterol, which account for > 84% (mol/mol) of the total lipid content. Table S1 shows the overall results. Figure S1 illustrates how the optimized Lactate flux is affected by the biomass composition. The prediction of the specific growth rate is not significantly affected (CV of 0.35%), in line with the conclusions presented by Széliová et al. [2]. The most affected byproducts are Lac, Pyr and Cit with CVs higher than 70%. However, their flux range is very low in OptCHO. Also Glu and Trp have a CV of 32.1% and 15.1% respectively. We thus conclude that the biomass composition does not significantly affect specific growth predictions and that only the low flux range values are significantly influenced by the biomass composition.

**Figure S1.** OptCHO Lac flux obtained for different biomass compositions. OptCHO calculations were repeated 100 times with random modifications of the biomass reaction coefficients of amino acids and selected lipids in the interval ±20% mol/gDW

**Table S1.** Influence of biomass composition in the OptCHO exchange fluxes. OptCHO calculations were repeated 100 times with random modifications of the biomass reaction coefficients of amino acids and selected lipids in the interval ±20% mol/gDW

| **Name** | **OptCHO** | **Mean** | **SD** | **CV** |
| --- | --- | --- | --- | --- |
| µ | 2,95E-02 | 2,95E-02 | 1,02E-04 | 0,347 |
| Glc | -3,30E-01 | -3,34E-01 | 1,44E-02 | 4,32 |
| Lac | -5,19E-03 | -4,42E-03 | 3,29E-03 | **74,3** |
| Gln | -1,02E-01 | -1,00E-01 | 5,98E-03 | 5,97 |
| Glu | -3,98E-03 | -3,65E-03 | 1,17E-03 | **32,1** |
| Nh4 | 6,83E-03 | 6,83E-03 | 7,27E-18 | 0,00 |
| Pyr | -5,29E-03 | -6,58E-03 | 4,81E-03 | **73,1** |
| Glyc | 5,07E-03 | 5,14E-03 | 2,26E-04 | 4,39 |
| Cit | 3,61E-03 | 4,48E-03 | 3,23E-03 | **72,1** |
| Ala | 8,60E-02 | 8,70E-02 | 3,96E-03 | 4,55 |
| Arg | -7,28E-03 | -7,30E-03 | 1,13E-04 | 1,55 |
| Asn | -5,50E-02 | -5,53E-02 | 1,09E-03 | 1,97 |
| Asp | -5,35E-03 | -5,23E-03 | 4,20E-04 | 8,04 |
| Lcystin | -1,89E-03 | -1,93E-03 | 1,27E-04 | 6,60 |
| Gly | 1,63E-02 | 1,64E-02 | 5,53E-04 | 3,36 |
| His | -3,48E-03 | -3,49E-03 | 5,76E-05 | 1,65 |
| Ile | -1,22E-02 | -1,23E-02 | 3,07E-04 | 2,50 |
| Leu | -2,36E-02 | -2,36E-02 | 2,07E-04 | 8,75 |
| Lys | -1,32E-02 | -1,31E-02 | 3,63E-04 | 2,77 |
| Met | -3,40E-03 | -3,40E-03 | 3,24E-05 | 9,53 |
| Phe | -5,66E-03 | -5,68E-03 | 1,09E-04 | 1,92 |
| Pro | -7,04E-03 | -6,95E-03 | 2,97E-04 | 4,27 |
| Ser | -2,45E-02 | -2,47E-02 | 9,37E-04 | 3,80 |
| Thr | -7,57E-03 | -7,60E-03 | 1,29E-04 | 1,70 |
| Trp | -1,31E-03 | -1,37E-03 | 2,07E-04 | **15,1** |
| Tyr | -3,72E-03 | -3,74E-03 | 1,21E-04 | 3,23 |
| Val | -1,36E-02 | -1,37E-02 | 3,59E-04 | 2,62 |

## **Toy example**

The objective of this toy example is to simulate a data set with a hidden mechanism and then to use this data set to compare the standard FBA and HybridFBA predictive power in the context of mechanistic uncertainty. The toy metabolic network represented in Figure S2 was adopted. This very simple network has m=5 intracellular species {A,B,C,D,E} and q=7 metabolic fluxes v={v_1_, v_2_, v_3_, v_4_, v_5_, v_6_, v_7_} of which 4 are exchange fluxes {v_1_, v_4_, v_6_, v_7_}. All reactions are irreversible, thus v_i_ ≥ 0 for i=1,…,7.


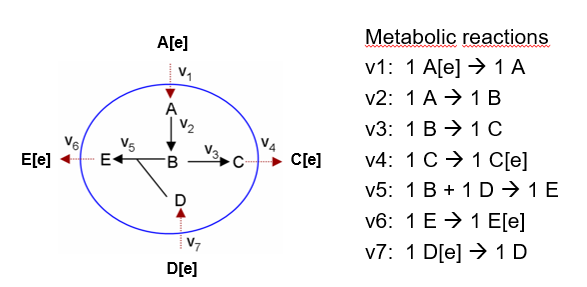


**Figure S1.** Toy metabolic network

The intracellular,
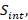
 and extracellular,
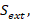
 stoichiometric matrices are as follows:


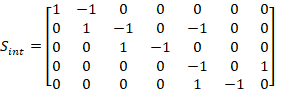
 (Eq. S2)


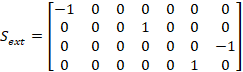
 (Eq. S3)

A set of 21 experiments were simulated by setting the exchange flux v_1_ to a fixed value between 0-10. The following hypothetical regulatory rule (this rule was hidden to the FBA/HybridFBA calculations) was used for the experiments simulation:

v5 = 0.5 × v2 (Eq. S4)

Given that the rank of
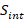
 is 5, the system becomes determined and unique solutions are obtained. The intracellular and exchange fluxes were calculated and registered in table S2. The exchange fluxes were corrupted by 20% gaussian noise.

**Table S2**: Simulation of 21 experiments with the toy network

| **Experiment** | **metabolic fluxes (noise free)** | | | | | | | **exchange fluxes (with 20% gauss. noise)** | | | |
| --- | --- | --- | --- | --- | --- | --- | --- | --- | --- | --- | --- |
|  | **v1** | **v2** | **v3** | **v4** | **v5** | **v6** | **v7** | **A[e]** | **C[e]** | **D[e]** | **E[e]** |
| **1** | 0,00 | 0,00 | 0,00 | 0,00 | 0,00 | 0,00 | 0,00 | 0,00 | 0,00 | 0,00 | 0,00 |
| **2** | 0,50 | 0,50 | 0,25 | 0,25 | 0,25 | 0,25 | 0,25 | -0,68 | 0,29 | -0,27 | 0,33 |
| **3** | 1,00 | 1,00 | 0,50 | 0,50 | 0,50 | 0,50 | 0,50 | -0,55 | 0,66 | -0,41 | 0,51 |
| **4** | 1,50 | 1,50 | 0,75 | 0,75 | 0,75 | 0,75 | 0,75 | -1,76 | 0,82 | -0,75 | 0,53 |
| **5** | 2,00 | 2,00 | 1,00 | 1,00 | 1,00 | 1,00 | 1,00 | -2,13 | 1,21 | -0,97 | 0,85 |
| **6** | 2,50 | 2,50 | 1,25 | 1,25 | 1,25 | 1,25 | 1,25 | -1,85 | 1,43 | -1,41 | 0,98 |
| **7** | 3,00 | 3,00 | 1,50 | 1,50 | 1,50 | 1,50 | 1,50 | -2,74 | 1,41 | -1,83 | 2,21 |
| **8** | 3,50 | 3,50 | 1,75 | 1,75 | 1,75 | 1,75 | 1,75 | -3,74 | 1,85 | -2,14 | 1,53 |
| **9** | 4,00 | 4,00 | 2,00 | 2,00 | 2,00 | 2,00 | 2,00 | -6,86 | 1,69 | -1,65 | 2,30 |
| **10** | 4,50 | 4,50 | 2,25 | 2,25 | 2,25 | 2,25 | 2,25 | -6,99 | 2,65 | -2,28 | 2,16 |
| **11** | 5,00 | 5,00 | 2,50 | 2,50 | 2,50 | 2,50 | 2,50 | -3,65 | 1,93 | -1,89 | 2,94 |
| **12** | 5,50 | 5,50 | 2,75 | 2,75 | 2,75 | 2,75 | 2,75 | -8,84 | 2,16 | -2,14 | 2,33 |
| **13** | 6,00 | 6,00 | 3,00 | 3,00 | 3,00 | 3,00 | 3,00 | -6,87 | 2,51 | -3,00 | 2,16 |
| **14** | 6,50 | 6,50 | 3,25 | 3,25 | 3,25 | 3,25 | 3,25 | -6,42 | 1,34 | -4,25 | 2,33 |
| **15** | 7,00 | 7,00 | 3,50 | 3,50 | 3,50 | 3,50 | 3,50 | -8,00 | 4,51 | -2,96 | 3,84 |
| **16** | 7,50 | 7,50 | 3,75 | 3,75 | 3,75 | 3,75 | 3,75 | -7,19 | 3,99 | -4,03 | 3,62 |
| **17** | 8,00 | 8,00 | 4,00 | 4,00 | 4,00 | 4,00 | 4,00 | -7,80 | 3,40 | -3,82 | 3,84 |
| **18** | 8,50 | 8,50 | 4,25 | 4,25 | 4,25 | 4,25 | 4,25 | -11,03 | 5,41 | -5,20 | 5,46 |
| **19** | 9,00 | 9,00 | 4,50 | 4,50 | 4,50 | 4,50 | 4,50 | -11,54 | 2,96 | -3,52 | 4,76 |
| **20** | 9,50 | 9,50 | 4,75 | 4,75 | 4,75 | 4,75 | 4,75 | -12,19 | 4,65 | -4,78 | 4,94 |
| **21** | 10,00 | 10,00 | 5,00 | 5,00 | 5,00 | 5,00 | 5,00 | -11,34 | 4,76 | -5,55 | 6,59 |

The “measured” exchange fluxes (last 4 columns in Table S2) were auto-scaled to zero mean and unit variance. The scaling factors were
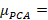
[-5.8178, 2.3633, -2.5161, 2.5812]^T^ and
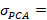
[ 3.9182, 1.5709, 1.6506, 1.8251] ^T^. The normalized rates were subject to PCA analysis with the 2 PCs (NPC=2) (Figure S2) resulting in the following (normalized) coefficients matrix:


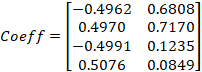
 (Eq. S5).

**Figure S2**: PCA analysis of the exchange flux data set (last 4 columns in Table S2). Left panel: explained variance by 2 PCs. Right panel: biplot of the 2^nd^ against 1^st^ rows of the
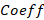
 matrix

Figure S2 shows that PC-1 explains 92% of the original data variance. We concluded that a single PC is sufficient to capture the data information content in this example. This was confirmed by simulating the data without experimental noise showing that a single PC captures100% of data variance.

Finally, standard FBA and HybridFBA were applied to maximize the flux v6 under the constraint of the exchange A[e] flux measurement. The standard FBA was computed with NPC=0. Given the PCA results above, HybridFBA was applied with NPC=1 (no calibration needed in this simple example). From the stoichiometric point of view, the system is undetermined with 1 degree of freedom (5 linearly independent equations and 6 unknown fluxes, given that v_1_ is measured). HybridFBA requires the information of the stoichiometric matrices (
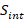
,
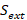
) and PCA coefficients (
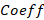
), which were inputted to the HybridFBA function. The objective function is set by the c coefficients as follows:


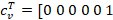
 0] (Eq. S6a)


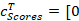
] (Eq. S6b)

The fluxes were constrained as follows (all reactions are irreversible; no upper bounds):


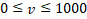
 (Eq. S7).

The scores (there is just 1 score of a single PC) were constrained as follows (in practice, unbounded):


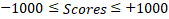
 (Eq. S8).

The exchange fluxes are left unbounded:


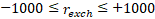
 (Eq. S9)

except for the exchange flux of A[e], which is bounded by a known value as follows:


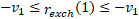
 (Eq. S10).

The optimization was performed for each experiment in Table S2 by setting the respective
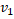
 values in Eq. (S10). To note that the regulatory rule (Eq. S4) is hidden to the FBA/HybridFBA, representing an unknown mechanism. The results are shown in Figs. S3-S5. Fig. (S3) shows that standard FBA failed to predict the unmeasured metabolic fluxes (v_2_-v_7_). Figs. (S4) (20% of gaussian noise) and (S5) (no noise) show that HybridFBA is able to perfectly describe the system depending on the level of noise in the data. With 20% gaussian noise an off-set in flux predictions is observed, which may be mitigated with additional data points.

**Figure S3**: Standard FBA flux prediction results (v_1_-v_7_) for all the experiments of Table S2 (Gaussian noise of 20%).

**Figure S4**: Hybrid FBA flux prediction results (v_1_-v_7_) for all the experiments of Table S2 (Gaussian noise of 20%).

**Figure S5**: Hybrid FBA flux prediction results (v_1_-v_7_) for all the experiments of Table S2 (without Gaussian noise).

The MATLAB code of this toy example is provided in the file HybridFBA_matlab.zip.

# **bibliography**

1- Ozturk, SS., Palsson, B. Ø. (1990). Chemical decomposition of glutamine in cell culture media: effect of media type, pH, and serum concentration. Biotechnol Prog, 6 (2), 121–128. doi: 10.1021/bp00002a005

2- Széliová, D., Ruckerbauer, D., Galleguillos, S., et al. (2020). What CHO is made of: variations in the biomass composition of Chinese hamster ovary cell line. Metab Eng, 61, pp. 288-300. doi:10.1016/j.ymben.2020.06.002

3- Hefzi, H., Ang, K. S., Hanscho, M., et al. (2016). A Consensus Genome-scale Reconstruction of Chinese Hamster Ovary Cell Metabolism. Cell Syst; 3(5), 434–443.e8. doi: 10.1016/j.cels.2016.10.020

4- Quek, L. E., Dietmair, S., Hanscho, M., et al. (2014). Reducing Recon 2 for steady-state flux analysis of HEK cell culture. J Biotechnol, 184, 172-8. doi:10.1016/j.jbiotec.2014.05.021

5- Schellenberger, J., Lewis, N. E., Palsson, B. Ø. (2011). Elimination of thermodynamically infeasible loops in steady-state metabolic models. Biophys J, 100(3), 544–553. doi: 10.1016/j.bpj.2010.12.3707

6- Heirendt, L., Arreckx, S., Pfau, T. et al. Creation and analysis of biochemical constraint-based models using the COBRA Toolbox v.3.0. (2019). Nat Protoc, 14, 639–702. doi:10.1038/s41596-018-0098-2
